# RORγt inhibition selectively targets IL-17 producing iNKT and γδ-T cells enriched in Spondyloarthritis patients

**DOI:** 10.1038/s41467-018-07911-6

**Published:** 2019-01-02

**Authors:** Koen Venken, Peggy Jacques, Céline Mortier, Mark E. Labadia, Tine Decruy, Julie Coudenys, Kathleen Hoyt, Anita L. Wayne, Robert Hughes, Michael Turner, Sofie Van Gassen, Liesbet Martens, Dustin Smith, Christian Harcken, Joseph Wahle, Chao-Ting Wang, Eveline Verheugen, Nadia Schryvers, Gaëlle Varkas, Heleen Cypers, Ruth Wittoek, Yves Piette, Lieve Gyselbrecht, Serge Van Calenbergh, Filip Van den Bosch, Yvan Saeys, Gerald Nabozny, Dirk Elewaut

**Affiliations:** 1 Department of Rheumatology, Faculty of Medicine and Health Sciences, Laboratory for Molecular Immunology and Inflammation, Ghent University, C. Heymanslaan 10, 9000 Ghent, Belgium; 2 VIB Inflammation Research Center, Ghent University, Technologiepark 71, 9052 Ghent, Belgium; 3 Research and Development Boehringer-Ingelheim, 175 Briar Ridge Road, Ridgefield, CT 06877 USA; 4 Department of Applied Mathematics, Computer Science and Statistics, Ghent University, Technologiepark 71, 9052 Ghent, Belgium; 5 AZ SintJan, Ruddershove 10, 8000 Brugge, Belgium; 6 ASZ Aalst, Merestraat 80, 9300 Aalst, Belgium; 7 Laboratory for Medicinal Chemistry, Faculty of Pharmaceutical Sciences, Ghent University, Ottergemsesteenweg 460, 9052 Gent, Belgium

## Abstract

Dysregulated IL-23/IL-17 responses have been linked to psoriatic arthritis and other forms of spondyloarthritides (SpA). RORγt, the key Thelper17 (Th17) cell transcriptional regulator, is also expressed by subsets of innate-like T cells, including invariant natural killer T (iNKT) and γδ-T cells, but their contribution to SpA is still unclear. Here we describe the presence of particular RORγt^+^T-bet^lo^PLZF^−^ iNKT and γδ-hi T cell subsets in healthy peripheral blood. RORγt^+^ iNKT and γδ-hi T cells show IL-23 mediated Th17-like immune responses and were clearly enriched within inflamed joints of SpA patients where they act as major IL-17 secretors. SpA derived iNKT and γδ-T cells showed unique and Th17-skewed phenotype and gene expression profiles. Strikingly, RORγt inhibition blocked γδ17 and iNKT17 cell function while selectively sparing IL-22^+^ subsets. Overall, our findings highlight a unique diversity of human RORγt^+^ T cells and underscore the potential of RORγt antagonism to modulate aberrant type 17 responses.

## Abstract (web summary)

The role of innate T cell subsets in the pathogenesis of spondyloarthritis (SpA) is not well understood. Here, the authors examine the role of invariant natural killer T (iNKT) and γδ-T cells in SpA and show that disease-derived iNKT and γδ-T cells have unique and Th17-skewed phenotype and gene expression profiles within inflamed joints.

## Introduction

Spondyloarthritides (SpA) refers to a cluster of inflammatory rheumatic diseases including ankylosing spondylitis (AS) and psoriatic arthritis (PsA), affecting nearly 1–2% of the Western population. Typical disease manifestations consist of inflammation of sacroiliac joints, as well as of the spine. Peripheral joints can also be affected (peripheral arthritis), as well as insertion of tendons to bone (enthesitis)^1^. Importantly, the disease also causes inflammation and tissue damage beyond the musculoskeletal system with the most typically affected organs being the eye (anterior uveitis), skin (psoriasis), and gut (inflammatory bowel disease—IBD). This association is further substantiated by a significant overlap in the underlying genetic predisposition for these disorders^2^. While the role for TNFα as therapeutic target in SpA has been documented widely, an emerging role for the IL-23/IL-17 inflammatory axis in this disease has arisen as evidenced by the marked efficacy of IL-17a inhibition (AS and PsA), as well as IL-12 and/or IL-23 blockade (PsA)^3–5^. Curiously, IL-17 inhibition failed to demonstrate efficacy in patients with rheumatoid arthritis (RA) and IBD^6,7^. IL-23 and IL-17 show divergent roles on epithelial barrier integrity in experimental models of gut inflammation^8,9^, which could explain the dichotomy of clinical efficacy of anti-IL-17 vs. IL-12/IL-23 inhibition in IBD patients, even though both are efficacious on joint symptoms in SpA. IL-22, the other IL-23 signature cytokine, also plays a key role in the maintenance of mucosal homeostasis by promotion of antimicrobial immunity, inflammation, and tissue repair at barrier surfaces^10^. However, it is not completely understood how differential expression of IL-17 and IL-22 is regulated and potentially disturbed at the cellular level in SpA^3^.

IL-23 is necessary for the terminal differentiation and inflammatory functions associated with T helper-17 cells (Th17), characterized by the expression of the key transcription factor retinoic acid receptor-related orphan receptor-yt (RORγt; which is encoded by *RORC*)^11^. Therefore, blockade of RORγt activity may constitute an alternative to antibody-mediated cytokine and/or receptor neutralization in treating IL-17-related diseases with a potentially broader mode of action^12^. In addition to conventional Th17 cells, other immune cells showing an innate(-like) phenotype, such as innate lymphoid cells (ILC), subsets of γδ-T cells, mucosal associated invariant T (MAIT) cells and invariant natural killer T cells (iNKT) also express RORγt and IL-23 signature cytokines^13^. Sherlock et al. showed that a specific subset of CD4-CD8-unconventional T cells in mice (which are most likely γδ-T cells) was able to drive SpA-like pathology upon induced systemic overexpression of IL-23, independent of Th17 cells^14^. This suggested that innate-like T cells may influence diseases like SpA^15^, because they display plasticity, and could therefore be skewed from an immunoprotective role towards a predominant IL-17 producing phenotype due to uncontrolled IL-23 signaling events. iNKT and γδ-T cells are subpopulations of innate-like T cells, which can release a broad spectrum of cytokines, both upon T cell receptor (TCR) activation and after TCR-independent stimulation, in this way acting as a ‘bridge’ between innate and adaptive immune responses. iNKT cells are characterized by the expression of a semi-invariant TCR (invariant Vα24-Jα18 in humans) associated with a restricted set of Vβ chains (Vβ11) and they respond towards glycolipids, including microbial α-glycuronylceramides and self-antigens, presented by the non-polymorphic MHC class I-like molecule CD1d^16^. iNKT cells can be separated into three distinctive subsets, iNKT1 cells, iNKT2 cells, and iNKT17 cells, analogous to the helper T cell lineage nomenclature^17^. γδ-T cells show no distinct MHC class restriction and are reactive towards both peptide (e.g., MHC related Ag such as MICA) and non-peptide (e.g., isoprenoid metabolites) antigens, by which they act as sensors of cellular stress and infection danger^18^. Moreover, they appear to be major IL-17 producers in the initial phase of many bacterial infections^19,20^. How human innate-like T cells such as iNKT and γδ-T cells are involved in inflammatory responses in arthritic disease, particularly SpA, is unclear and whether these cells can be skewed by pharmacological modulation is equally unknown.

Here we identify novel innate-like T cell subset—RORγt^+^T-bet^lo^PLZF^−^ iNKT and γδ-hi T cells—circulating in healthy peripheral blood and clearly expanded in inflamed joints, posed to rapidly respond to IL-23 by steering Th17-like immune responses. By using FlowSOM^21^, we show a previously unappreciated heterogeneity of human iNKT and γδ T cells of which specific subsets are abnormally represented in the peripheral blood of SpA patients. RNASeq analyses revealed a markedly distinct gene signature profile consistent with bias towards distinct IL-17 pathway regulation in innate-like T cells from SpA vs. RA patients. Finally, pharmacologic modulation using RORγt inhibition blocked effector function of disease associated subsets while preserving IL-22 subsets, underscoring the potential of RORγt antagonism to modulate aberrant type 17 responses.

## Results and discussion

### Human blood RORγt^+^ iNKT and γδ cells lack expression of PLZF

**Fig. 1:**
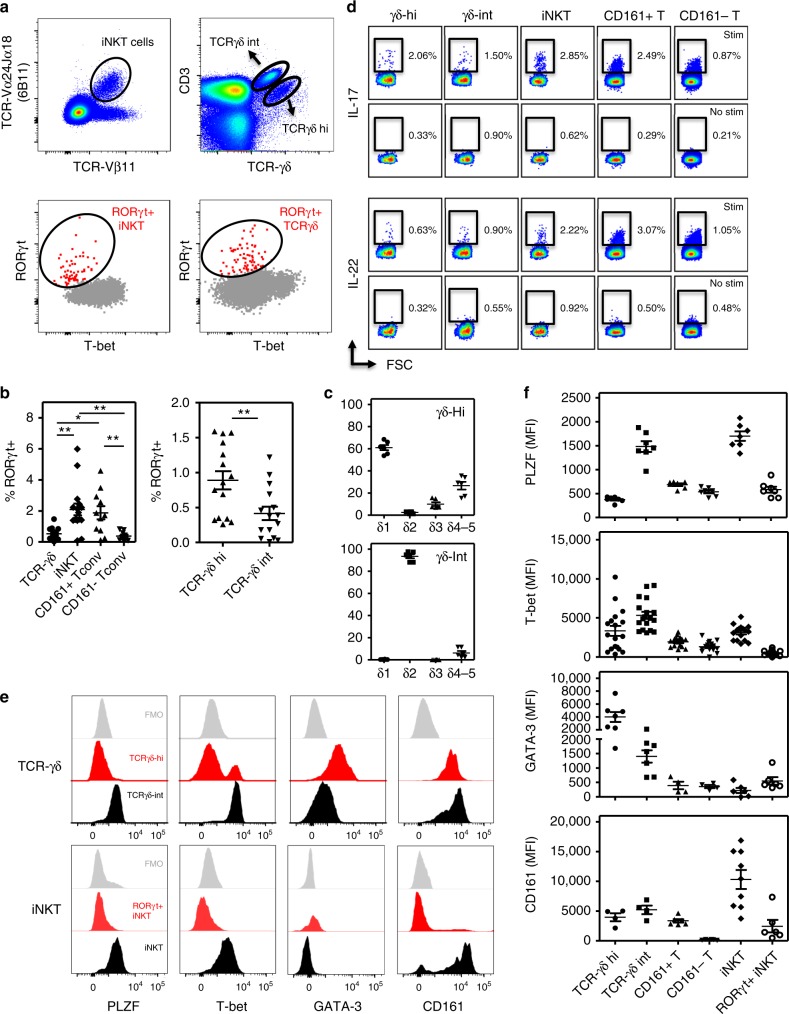
Human blood circulating RORγt+ iNKT and γδ-T cells lack expression of PLZF. **a** Flowcytometric analyses showing distinct subsets of blood circulating RORγt^+^T-bet^lo^ iNKT and total γδ-T cells (flow plots show representative data from a healthy individual). Gating strategy is shown in Supplementary Fig. 1A. **b** Percentages of RORγt^+^T-bet^lo^ cells in iNKT (*n* = 16) and γδ-T cells (*n* = 15) and CD161+ and CD161- Tconv (*n* = 11) (left panel; *p < 0.05, **p < 0.01 as determined by ANOVA) and in γδ-T cell subsets (right panel; paired *t*-test). **c** TCRVδ profiling of indicated γδ-T cells (*n* = 6). Representative flow plots are shown in Supplementary Fig. 1B (**d**) IL-17 and IL-22 production by indicated subsets as measured by intracellular cytokine staining after 4 h incubation of PBMC with PMA/CaI/BFA (stim) or BFA alone (no stim). Results from one experiment are shown (representative for *n* = 6). Summary data are presented in Supplementary Fig. 1C. **e** Stacked histograms showing expression levels of PLZF, T-bet, GATA-3, and CD161 measured in iNKT and γδ-T cells (one representative example). **f** MFI values for indicated markers (as indicated in **d**). Each dot represents data from one healthy individual (PLZF and GATA-3 *n* = 7; CD161 *n* = 9, T-bet *n* = 16). Overview of statistics (ANOVA) is provided in the Supplementary Table 3. Data throughout this figure are presented as mean ± SEM

Murine RORγt ^+^ IL-17 producing iNKT (also denominated iNKT17) and γδ-T cells (γδ17) have been extensively studied but the prevalence and phenotype of their human counterparts is poorly understood. We could delineate rare subsets of RORγt^+^T-bet^lo^ iNKT (6B11+) and γδ-T cells in the blood of healthy individuals (Fig. 1a and Supplementary Fig. 1A). RORγt^+^T-bet^lo^ cells represented on average 2.1% (0.09–6%) of total iNKT cells, comparable to frequencies of RORγt^+^T-bet^lo^ cells (1.9 ± 1.4%; mean ± SEM %) detected in CD161 + Tconv cells, a population enriched in Th17 cells^22^. The prevalence of RORγt^+^T-bet^lo^ in the γδ-T population was less frequent (0.5 ± 0.4%), but still relatively increased compared to kindred cells in the CD161- Tconv population (Fig. 1b). Interestingly, based on TCR/CD3 expression levels, we could distinguish two populations of γδ-T cells, a large fraction of TCRγδ-int cells and a minor subset of TCRγδ-hi cells, with the latter showing a significant enrichment of RORγt^+^T-bet^lo^ cells (Fig. 1a, b). Phenotypical different subsets of murine γδ-T cells have also been discriminated based on TCR expression levels and/or γδ gene usage^23–25^. Moreover, CD3^bright^ γδ-T cells showing the canonical Vγ6/Vδ1 + TCR have been correlated with the γδ17 phenotype and these cells are preferentially found in lung and skin tissues of mice^25^. With regard to TCR gene usage/TCR-δ repertoire of human γδ subsets shown here, it was clear that the majority of TCRγδ-int cells belonged to the δ2 TCR subfamily (being Vγ9Vδ2), whereas TCRγδ-hi cells are enriched in δ1 cells although they also included a minor subset of δ3 cells (Fig. 1c and Supplementary Fig. 1B). The capacity of iNKT and these γδ-T cells to secrete IL-17 and to a lesser extent IL-22 cytokines (directly ex-vivo after short term PMA/CaI stimulation), was consistent to their RORγt expression profile (Fig. 1d and Supplementary Fig. 1C).

In contrast to total iNKT and TCRγδ-int T cells, RORγt^+^T-bet^lo^ iNKT and TCRγδ-hi T cells showed very low levels of promyelocytic leukemia zinc finger (PLZF) (Fig. 1e, f and Supplementary Fig. 1D). These findings could also be confirmed when using αGalCer-hCD1d tetramers for detection of iNKT cells (Supplementary Fig. 1e, f). In mouse models it has been shown that PLZF plays an important role in the early development of iNKT cells^26,27^ and PLZF expression has also been identified in MAIT cells^28^, innate subsets of γδ T cells^29^ and fetal ILC^30^, suggesting a determinant role for PLZF in the common innate phenotype of these cells. In humans, also NK cells and small subsets of CD4 and CD8 T cells seem to express PLZF^31^, highlighting substantial differences in the importance of PLZF gene expression between mice and humans. The low PLZF expression in human blood iNKT17 cells seems to contrast observations that PLZF regulates the acquisition and maintenance of a human Th17 phenotype^32^. However, recent studies showed that iNKT cells have a unique transcriptional profile distinct from that of NK cells and MHC-restricted T cells^33^. Moreover, the transcriptional nature of iNKT17 cells was similar to analogous subsets of γδ-T cells and ILC cells, while not to Th17 cells^34^. A possible explanation could be that human RORγt^+^ iNKT cells develop as a distinct subset of PLZF^neg^ cells in the thymus. At least in mice this scenario seems unlikely, as thymic iNKT17 cells express intermediate levels of PLZF, in between those measured in iNKT1 (PLZF^lo^) and iNKT2 (PLZF^hi^) cells^17^. Alternatively, iNKT17 cells might lose PLZF expression upon peripheral migration and/or activation and differentiation. In this regard, PLZF^lo^ iNKT cells have been identified in murine adipose tissue, not linked to RORγt expression or IL-17 production but to an anti-inflammatory IL-10 driven phenotype, indicating a specific local role for these PLZF^lo^ iNKT cells in adipose tissue immunoregulation^35^. In mice, iNKT17 cells are very scarce in peripheral circulation and the majority of iNKT17 cells seem to be tissue resident cells that can be found in lung tissue and lymph nodes^36^. iNKT subsets are uniquely distributed in peripheral lymphoid and non-lymphoid organs with some inter-strain variation^36^. They do not (or only modestly) recirculate, but instead show very long-term residence at these locations^35,37^. Our data suggest that human peripheral blood RORγt^+^T-bet^lo^ iNKT cells might show an intermediate/transitional phenotype of iNKT17 cells, prior to migration of these cells into various tissues, where they will further differentiate under influence of the local microenvironment and signaling cues.

Most work on human γδ-T cells has focused on Vγ9Vδ2 T cells, which form the major population of blood γδ-T cells. Of note, this subset is not found in mice due to the absence of orthologous TCR gene segments^18^. We found that γδ-T cells contain two phenotypically distinct subsets based on TCR expression levels, with TCRγδ-hi cells being predominantly PLZF^+^, GATA-3^+^, and Tbet^lo^. Although the data on the role of PLZF in γδ-T cells are more limited, they also point towards an innate differentiation program in these cells, as noticed for selective subsets of murine (Vγ1^+^Vδ6.3/Vδ6.4^+^) γδ-T cells^29,31,38^. In contrast, previous work indicated that PLZF is present in all human γδ-T cells^31^, but our data highlight this is especially true for the TCRγδ-int cell population, while TCRγδ-hi cells, containing augmented numbers of RORγt^+^ cells (Fig. 1b), are clearly PLZF^lo^. This seems in concordance with Kreslavsky et al. showing that thymic mouse γδ-T cells capable of producing IL-17 are PLZF negative^29^. However the majority of human PLZF^lo^ γδ-T cells did not express RORγt, so the precise relation between expression of these transcription factors in γδ-T cells needs further investigation.

Altogether, these data identify RORγt^+^ iNKT and γδ-hi T cells as a minor fraction of PLZF^neg^ innate-like T cells in the peripheral blood of healthy individuals.

### iNKT and γδ-T cells are armed for a competent IL-23 response

**Fig. 2:**
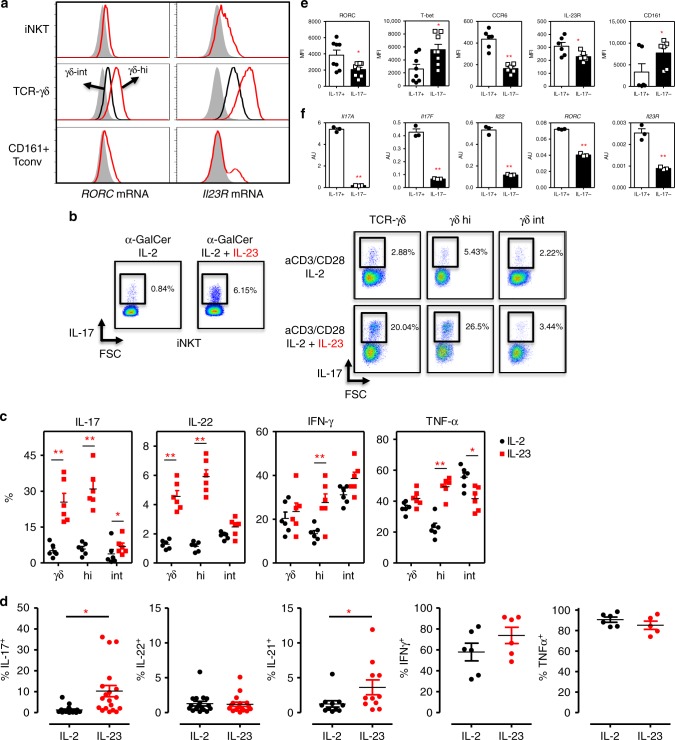
iNKT and γδ-T cells are armed for a competent IL-23 response. **a** *RORC* and *IL23R* mRNA transcripts expressed in iNKT, TCRγδ-hi/int, and CD161+ conventional T cells as measured by PrimeFlow technology (*n* = 3). Gray histograms represent FMO. Quantitative data are presented in Supplementary Fig. 2B. **b** PBMC (left) or sorted γδ-T cells were cultured with αGalCer (PBMC, *n* = 18) or aCD3/aCD28 Abs (γδ-T cells, *n* = 6), in the presence of IL-2 or IL-2 supplemented with IL-23, IL1β, TGFβ1 to induce IL-17 cytokine response as measured from viable iNKT (6B11+ TCRvβ11 + CD3+ cells) or γδ-T cells (TCRγδ+ CD3+). Flow data from one representative experiment is shown. **c**, **d** Quantitative overview of cytokine production measured in γδ-T cell (C; IL-2: black symbols; IL-23: red symbols) and iNKT (D, IL-2 condition: black symbols; IL-23: red symbols) cultures as described in **b** (IL-23 vs. IL-2 condition, *p < 0.05, **p < 0.01 determined by paired *t*-tests). **e** FACS analyses were performed on gated IL17+ and IL17− iNKT cells depicted in experiments described in **b** (*n* = 6–8). *p < 0.05, **p < 0.01 determined by paired t-tests (IL-17- cells: black bars; IL-17+: white bars). **f** qPCR assays on selected target genes, were performed on IL17+ and IL-17− iNKT cells isolated by means of a IL-17 Capture Assay (qPCR, *n* = 3, Mann-Whitney test). (IL-17- cells: black bars; IL-17+: white bars). Data throughout this figure are presented as mean ± SEM

As IL-23 and IL-23 receptor expression is clearly associated with Th17 pathogenicity^13^, we examined IL-23 responsiveness of human iNKT and TCRγδ T cell subsets. Consistent with data obtained from IL-23R reporter mice^39^, we noticed that surface IL-23R expression was almost completely absent on human blood derived iNKT and TCRγδ T cells (Supplementary Fig. 2A). In naïve mice, IL-23R is prominently found on lamina propria lymphocytes (LPL) γδ-T cells^39^, underscoring the importance for IL-23 at barrier sites. Using PrimeFlow technology, we measured low but significant amounts of *RORC* mRNA transcripts in iNKT, TCRγδ-int, and CD161+ conventional T cells, in contrast to high levels in TCRγδ-hi T cells (Fig. 2a and Supplementary Fig. 2B). Interestingly, significant IL-23 *receptor (IL-23R)* mRNA transcripts could be found in iNKT cells, a subset of CD161+ T cells and the majority of TCRγδ T cells. In addition to differences in *RORC* mRNA, *Il-23R* mRNA was also expressed at higher levels in TCRγδ-hi as compared to TCRγδ-int cells further underscoring the phenotypical variance between these γδ-T cell subsets.

Next, we tested blood TCRγδ and iNKT cell function in response to IL-23 stimulation in vitro. As expected from the absence of significant surface IL-23R expression, short term stimulation of sorted iNKT or TCRγδ T cells with IL-23 alone did not lead to IL-17 release by any cell type (data not shown). However, upon culturing iNKT or TCRγδ T cells with a TCR stimulus (aGalCer or αCD3/CD28Ab, respectively), we found a strong enrichment of IL-17 producing cells in those cultures where we added IL-23 as compared to IL-2 alone (Fig. 2b), in agreement with published data^20,40^. TCRγδ-hi cells showed an augmented capacity to produce IL-17 and also IL-22 as compared to TCRγδ-int cells upon IL-23 priming (Fig. 2b, c). TCRγδ-hi cells producing IFNγ and TNFα were also induced by IL-23, matching the frequencies found in TCRγδ-int cells. Parallel to induction of IL-17, IL-23 skewed iNKT cells contained significantly increased numbers of IL-21^+^ cells, whereas the frequency of IL-22, TNFα and IFNγ producing cells was comparable between the IL-2 and IL-23 conditions (Fig. 2d). The phenotypical profile of IL-23 induced IL-17+ iNKT cells was further evaluated by means of flow cytometry and qPCR analyses on sorted IL-17^+^ cells as obtained by a cytokine capture assay (Fig. 2e, f). These data confirmed that IL-23 induced iNKT17 cells showed higher levels of RORC and lower levels of T-bet expression and CD161 as compared to IL-17^neg^ counterparts (Fig. 2e and Supplementary Fig. 2C), mimicking the ex-vivo phenotype (Fig. 1d). Moreover, iNKT17 cells also expressed higher levels of CCR6 a chemokine receptor associated with Th17 cells, and contained high levels of *IL17A*, *IL17F*, and also *IL22* mRNA (Fig. 2f).

Overall, these data indicate that human circulating iNKT cells and especially γδ-hi T cells show significant levels of *IL23R* mRNA transcripts which enables them to rapidly induce Th17-like immune responses.

### iNKT and γδ-T cells in SpA joints show a IL-17 signature

**Fig. 3:**
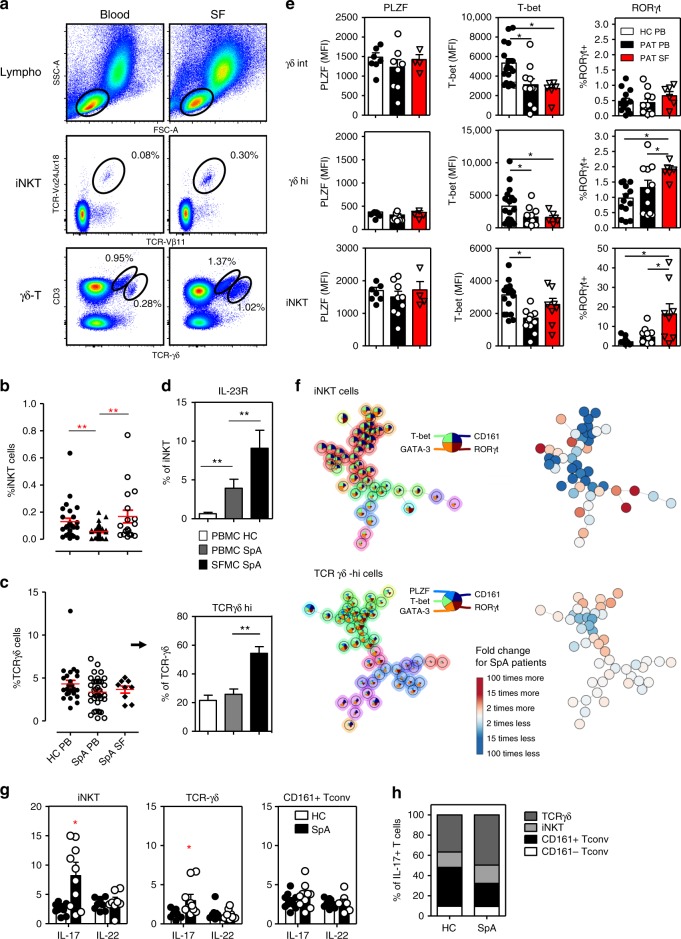
iNKT and γδ-T cells in SpA joints show a profound IL-17 signature. **a** Flowcytometric analyses of iNKT and γδ-T cells in paired PBMC and SFMC derived from a representative SpA patient. **b** Mean percentage (±SEM) of iNKT cells (relative to total CD3+ T cells) measured in PBMC (SpA, *n* = 33; HC, *n* = 27) and SFMC (SpA, *n* = 18) samples (**p < 0.01 as determined by ANOVA). **c** Frequency of γδ-T cells and TCRγδ-hi cells (relative to total CD3+ T cells and γδ-T cells, respectively) in PBMC and SFMC samples (ANOVA). **d** Mean percentage IL-23R^+^ cells (relative to iNKT cells) measured in PBMC and SFMC samples from B (ANOVA). **e** Transcriptional factor profile of iNKT cells and γδ-T cells measured in PBMC and SFMC from SpA patients (PB *n* = 8-12, SF *n* = 4-8) and HC (*n* = 8-16). (*p < 0.05 as determined by ANOVA) (HC-PB: white bars, SpA-PB: black bars, SpA-SF: Red bars). **f** FlowSOM visualization of indicated immune cell population present in the peripheral blood of HC (*n* = 8) and SpA patients (*n* = 8), stained for markers indicated in the pie chart. Each circle represents a specific marker combination, corresponding to a specific cell type. The mean marker values are visualized for each node, using star charts. The height of each part indicates the expression intensity: if the part reaches the border of the circle, the cells have a high expression for that marker. The nodes are connected to the ones they are the most similar to. An automatic meta-clustering of the FlowSOM nodes is visualized by the background color of groups of nodes. A parallel tree gives an overview of significantly over-(red) or under-(blue) represented cell types in SpA patients vs. controls. **g** IL-17 and IL-22 production by peripheral blood iNKT and γδ-T cells from SpA patients (*n* = 10; black bars) and controls (*n* = 10, white bars) as measured by intracellular cytokine staining after 4 h incubation of cells with PMA/CaI in the presence of BFA (*p < 0.05, *t*-tests HC vs. SpA). **h** Relative representation of indicated peripheral blood T cell subsets among IL-17 producing T cells as measured in PMA/CaI stimulated PBMC from HC and SpA patients (see Supplementary Fig. 6A for individual data points and statistics). Data throughout this figure are presented as mean ± SEM unless stated otherwise

Because recent data point to the importance of IL-23/IL-17 driven inflammation in mediating disease features in SpA, we examined RORγt^+^ innate-like T cells in blood and synovial fluid (SF) from new onset SpA patients. We first analyzed overall frequencies of iNKT and TCRγδ T cell subsets in paired peripheral blood and SF samples from patients with active joint swelling. The frequency of blood circulating iNKT cells in SpA was significantly reduced vs. age matched healthy controls (Fig. 3a, b; HC 0.130 ± 0.025% vs. SpA 0.051 ± 0.008%; *p* < 0.01, ANOVA). By contrast, iNKT cell numbers were increased in the SF samples of these patients (0.168 ± 0.047%; *p* < 0.01 as compared to SpA blood). Frequencies of γδ-T cells were not significantly reduced in circulation of SpA patients compared to healthy controls (Fig. 3c; HC 4.31 ± 0.41% vs. SpA 3.34 ± 0.27%; *p* > 0.05, ANOVA). But curiously, in SF of SpA patients the prevalence of especially the TCRγδ-hi subset was remarkable augmented compared to peripheral blood (Fig. 3c; SpA blood 25.8 ± 3.6% vs. SF 54.3 ± 4.7% of γδ cells; *p* < 0.01, ANOVA). These alterations in iNKT and γδ-T cells were also reflected in patient derived synovial tissue samples (inflamed knee) (Supplementary Fig. 3). Moreover, there was also a significant increase in the number of IL-23R^+^ iNKT and γδ-T cells in the joint compartment of SpA patients (Fig. 3d; iNKT: blood 4.3 ± 1.2% vs. SF 9.0 ± 2.2%; Fig. 5c γδ-T: blood 4.6 ± 1.7% vs. SF 17.9 ± 13.9% of γδ-T cells; both *p* < 0.01, ANOVA).

A hallmark of innate-like T cells such as iNKT and γδ-T cells is their functional plasticity which is reminiscent, and may even exceed, that of conventional CD4 T cells^3^. We therefore analyzed the transcription factors controlling effector functions in these subsets in the blood and joints of SpA patients. RORγt^+^ cells in iNKT and to lesser extent TCRγδ-hi, were significantly enriched in SF vs. blood and accompanied with a decrease in T-bet levels. T-bet levels were also significantly reduced in circulating iNKT cells and γδ-T cells of SpA patients compared to controls (Fig. 3e). In contrast, no significant differences were found in PLZF expression (Fig. 3e). The flow cytometry dataset generated from the patient and control samples was further evaluated by FlowSOM, a novel and highly efficient tool for unsupervised clustering of cell types^21^. Traditional, linear analyses of multi-parameter flow cytometry data can suffer from limitations and the recent progress in computational methods has led to novel insights into immune cell diversity^41^. Human peripheral blood iNKT and γδ-T cell subsets were visually represented in a minimal spanning tree, where each node (circle) corresponds to a group of phenotypically related cells, and neighboring circles represent more similar cell types (Fig. 3f). FlowSOM metacluster analyses showed a marked heterogeneity of blood circulating iNKT and γδ-T cell subsets (based on their transcriptional factor profile). Moreover, we observed a skewing of particular innate-like T cell subsets in the blood of SpA patients, which was most pronounced for iNKT cells (Fig. 3f and Supplementary Fig. 4). SpA patient blood circulating conventional T cells showed a comparable FlowSOM profile with corresponding cells from control individuals, although some minor significant differences were observed for the CD161+ population (Supplementary Fig. 4 and 5). When we evaluated their cytokine production (directly ex-vivo after short term PMA/CaI stimulation), we observed increased numbers of IL-17^+^ cells in blood derived iNKT and TCRγδ but not Tconv cells from SpA patient vs. healthy controls (Fig. 3g; iNKT 2.81 ± 0.64% vs. 8.20 ± 2.27% IL-17^+^ ; γδ: 1.16 ± 0.21% vs. 2.97 ± 0.80% IL-17^+^ for HC vs. SpA, respectively; both *p* < 0.05, ANOVA). Moreover, innate-like T cells represent about half of all IL-17 producing blood circulating T cells, and this fraction is even higher in SpA (Fig. 3h and Supplementary Fig. 6A).

**Fig. 4:**
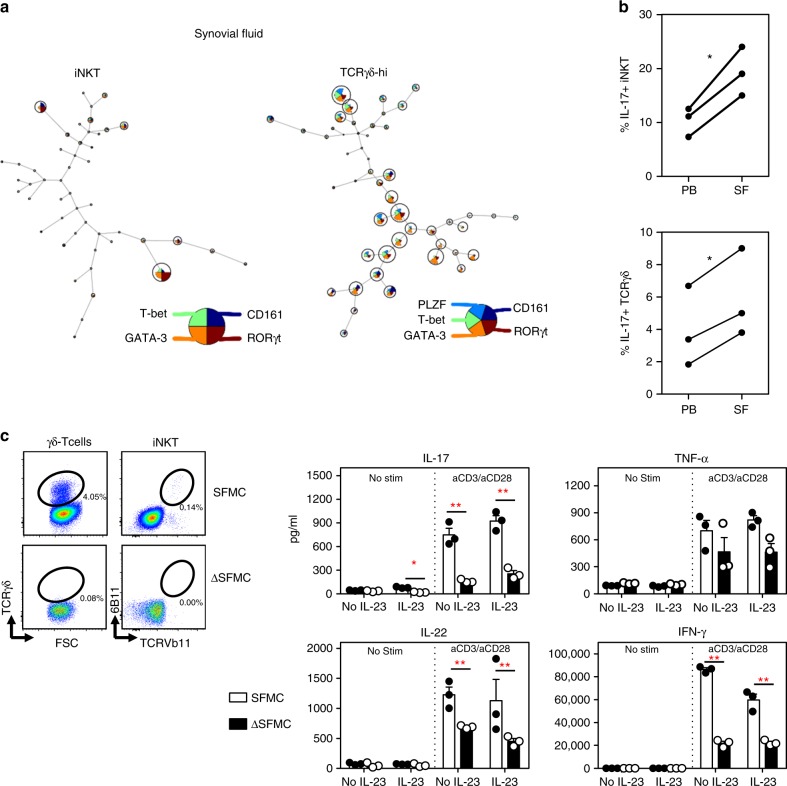
iNKT and γδ-T cells are a major source of T cell derived IL-17 in SpA joints. **a** FlowSOM visualization of iNKT and γδ-hi T cells present in synovial fluid samples from three treatment naïve SpA patients, stained for the indicated markers set (as explained in Fig. 3F). Nodes show cell subsets (including RORγt + cells) enriched in the synovial fluid (compared to cells from paired blood samples). Other subsets (nodes) are present but at a relatively lower frequencies. **b** Paired analyses of IL-17 production by SpA SF and blood derived iNKT and γδ-T cells as determined in Fig. 3G. (*p < 0.05, paired *t*-tests). **c** Synovial fluid derived mononuclear cells (SFMC) and SFMC cells depleted of iNKT and TCRγδ cells (ΔSFMC, illustrated in the plots) were cultured in the presence or absence of IL-23 combined with or without aCD3Ab/aCD28Ab stimulation. Supernatants were collected at 72 h of culture and cytokines production was measured by ELISA (SFMC vs. ΔSFMC; *p < 0.05, **p < 0.01 two-way ANOVA). Results from one experiment are shown (Representative for a total of three independent experiments using SF samples from 3 SpA patients). SFMC data (white bars), ΔSFMC (black bars). Data throughout this figure are presented as mean ± SEM

iNKT and γδ-T cells were found to be even further skewed towards RORγt and IL-17 expressing subsets in SF samples from SpA patients (Fig. 4a, b) as determined by FLOWSOM (Fig. 4a) and intracellular cytokine stainings (Fig. 4b). Moreover, elevated RORγt expression levels were also measured in synovial tissue residing iNKT and γδ-T cells (Supplementary Fig. 3). To assess the contribution of these innate-like T cells in IL-17 inflammation in the joints, we selectively depleted iNKT and γδ-T cells from SpA SF mononuclear cells (SFMC) and assessed the impact on cytokine production following TCR ligation. For this, we measured IL-17 and IL-22 (next to TNFα and IFNγ) secretion in cultures of SFMC in the presence or absence of iNKT and TCRγδ cells (SFMC and ΔSFMC, respectively). Both SFMC and ΔSFMC fractions were processed under identical conditions and stimulated with or without IL-23 and in the absence or presence of TCR stimulation (Fig. 4c). These experiments highlighted that the absence of iNKT/TCRγδ cells led to a marked decrease in IL-17, IL-22, and IFNγ production upon TCR ligation, a finding also observed under different stimulation conditions (Supplementary Fig. 6B). TNFα levels did not significantly change in ΔSFMC vs. SFMC cells (Fig. 4c). Interestingly, IL-23 alone also induced some IL-17 in SFMC but not in ΔSFMC cells (Fig. 4c) consistent with the observed IL-23R abundance on these cells in the joint compartment. These functional data provide clear evidence that innate-like T cells such as iNKT and γδ-T cells, although numerically not the most abundant immune cell subset, act as major T cell sources of IL-17, IL-22, and IFNγ in SpA joints.

Taken together, there is a previously unrecognized diversity of human innate-like T cells of which some RORγt^+^ subsets show a particular skewing in SpA patient blood and joint fluid. Thus, RORγt^+^ iNKT and γδ-T cells accumulate in joints of patients with SpA, and potentially together with other innate-like immune cells such as MAIT^42^ and ILC^43,44^ act as major contributors to IL-17 mediated pathology in SpA joints.

### iNKT and γδ-T cells show different gene expression profiles in SpA and RA

**Fig. 5:**
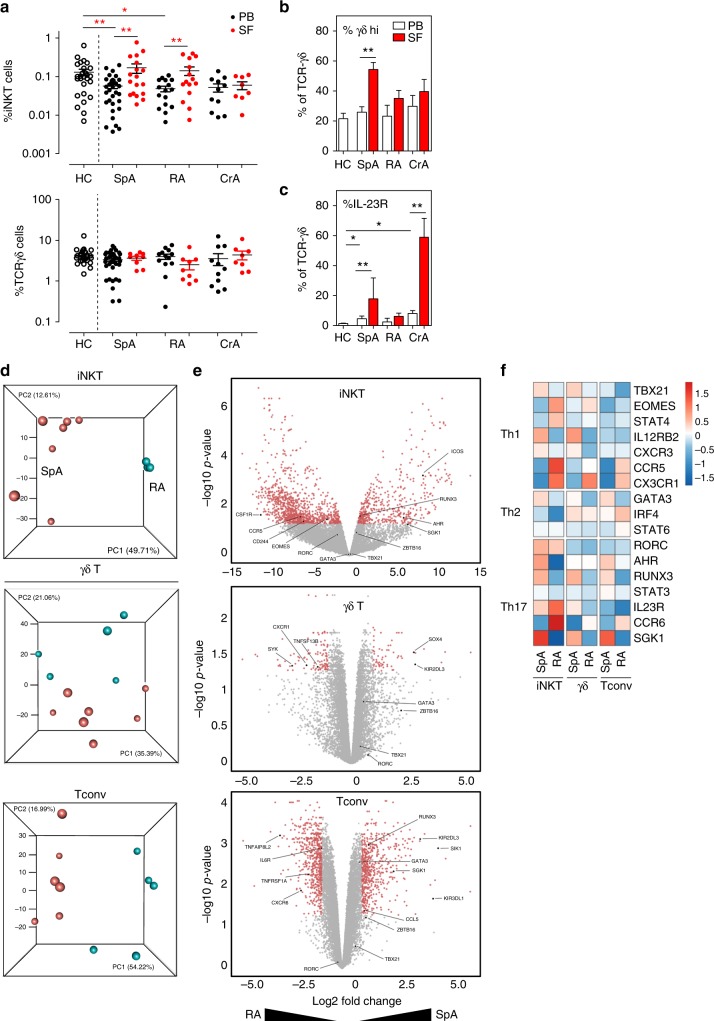
iNKT and γδ-T cells show different gene expression profiles in SpA and RA patients. **a** Frequencies of iNKT and γδ-T cells in indicated groups of rheumatic patients (each dot represents data from a single patient). Statistical analyses (*p < 0.05, **p < 0.01) referred to comparisons of blood samples (patients vs. controls) and comparisons of SF as compared to paired blood samples of patients as done by ANOVA and paired *t*-tests, respectively (same for **b** and **c**). (PB: black symbols; SF: red symbols) **b** Relative percentage of TCRγδ-hi cells and **c** IL-23R (lower panel) in γδ-T cells of samples taken from patients and controls. (PB: white bars; SF: red bars) **d** Principal component analysis (PCA) of transcriptomes (RNAseq data) of iNKT, γδ-T and Tconv cells sorted from SpA (*n* = 7, red) and RA patients (*n* = 5, blue). Each sphere symbol represents an individual patient. **e** Differentially expressed genes are represented in Volcano plots after pairwise comparisons between indicated subsets of SpA and RA patients. Each symbol represents a single gene and genes of interest are highlighted (see also main text). Color coding refers to differentially expressed genes with adjusted p-value less than 0.05 and the log2FC less than −1 (higher expressed in RA derived cells) or greater than 1 (higher expressed in SpA). **f** Heatmap showing relative expression Th1, Th2, and Th17 related genes in indicated cell subsets from SpA and RA patients. Data throughout this figure are presented as mean ± SEM

RA is a chronic autoimmune joint disease which has also been linked to Th17 mediated pathology^45^. Unexpectedly, inhibition of IL-17A with secukinumab, as showed in a phase 3 clinical trial, failed to demonstrate major clinical benefits^7^, which contrast with its broad efficacy in SpA^4^. We therefore evaluated whether the altered iNKT17 and γδ17 T cell profile we observed was a specific finding for SpA. We first evaluated iNKT and γδ-T cell numbers in blood and SF samples of treatment naïve RA patients in addition to patients with crystal induced arthritis (CrA, including gout/chondrocalcinosis), prototypic forms of acute –inflammasome/IL-1- driven arthritis^46^ (Fig. 5a). An enrichment of iNKT cells in peripheral joints was also observed in RA patients, but not in CrA patients Fig. 5a). There were no significant differences in total TCRγδ+ cell numbers in blood vs. SF samples from RA nor CrA patient and only SpA patients showed a significant increase in IL-23R^+^ γδ-hi cells in the joints (Fig. 5b, c). Notable, there was a subset of IL-23R^+^ γδ-T cells clearly present in the SF of CrA patients (Fig. 5c). This seems unrelated to observations in SpA, as these CrA patient γδ-T cells were not enriched in TCRγδ-hi cells. Whether these cells are potentially driven by uric acid crystal induced IL-1 signaling events needs additional study.

We sorted peripheral blood iNKT and TCRγδ T cells next to CD161^−^ Tconv cells from 7 SpA and 5 RA patients prior to gene expression profile analyses. Principal component analysis (PCA) of normalized transcriptomics data showed separate clustering of iNKT cells of RA patients and SpA patients and to a lesser extent also for γδ-T cells and Tconv cells indicating clear differences in gene expression profiles between SpA and RA patients (Fig. 5d). Next, differentially expressed genes in SpA vs. RA derived cells were evaluated and volcano plots were generated showing genes (red dots) at least 2 fold upregulated in either SpA or RA (adjusted *p* < 0.05) (Fig. 5e). Pairwise comparison of iNKT, γδ-T cells and Tconv of SpA and RA patients indicated 1594, 168, and 1206, respectively, differentially expressed genes. When comparing cells from SpA and RA patients, we detected no significant differences in expression of the Th cell signature transcription factors, *RORC (Th17), TXB21 (Th1) or GATA3 (Th2)*, for any of the investigated T cell subsets. Interestingly, for iNKT cells, there was clear upregulation of iNKT17 associated genes (*AHR, SGK1, RUNX3, and ICOS*) in SpA and upregulation of iNKT1 associated genes (*CD244, CSF1R, EOMES, CCR5*) in RA. The overexpression *AHR* and *SGK1* mRNA in SpA vs. RA immune cells are of interest as they are known to be triggered by environmental signals. Indeed, *AHR* (Aryl Hydrocarbon Receptor) encodes for a ligand-activated nuclear transcription factor which has been shown to be expressed by iNKT17 cells, although rather promotes IL-22 secretion while suppressing IL-17^40^. AHR activation is induced by naturally occurring compounds such as tryptophan metabolites (derived from microorganisms), next to synthetic polycyclic aromatic hydrocarbons and dioxin-like compounds^47^. *SGK1* (Serum/Glucocorticoid Regulated Kinase 1) gene expression in iNKT cells has not been described yet, but may be critically important as this kinase is involved in salt induced signaling processes and it is able to promote Th17 function while suppressing Treg function^48,49^. In addition, γδ-T cells of SpA patients differed substantially from RA by a remarkable upregulation of SOX4, a high-mobility group (HMG) box transcription factor, key in γδ17 cell-specific transcription program^50^, whereas we noted that *IL18RA* (*CXCR1*) and *BAFF* were significantly higher expressed in RA. BAFF, is a key cytokine for B cell activation and maturation, mainly expressed by myeloid cells and stromal cells, but it was recently also shown to be expressed by human TCRγδ cells upon TGF-β/IL-15 stimulation^51^. Finally, Heatmap visualization of typical Th cell genes demonstrated that innate-like T cell subsets, especially iNKT, to be more skewed towards a Th17 cell associated profile in SpA as compared to RA (Fig. 5f)

In addition to these marked differences in innate-like T cells, also conventional T cells displayed some marked differences between SpA and RA. This included expression of several Killer cell immunoglobulin-like receptors (KIRs), transmembrane glycoproteins expressed by natural killer cells and subsets of T cells, which were upregulated in SpA patients. For example, KIR3DL1 was reported to act as a receptor for HLA-B27 homodimers^52^ representing a mechanism by which HLA-B27 may be linked to aberrant NK and T cell responses in SpA^53^. By contrast, RA conventional T cells showed increased *IL6R* mRNA levels, as well as differences in expression of TNF signaling genes such as *TNFRSF1A* (encoding the TNFR1) and *TNFAIP8L2* (TIPE2), both previously linked to RA disease pathogenesis^54^.

Collectively our data provides evidence for a “type 17” skewed gene expression profile in SpA innate-like Tcells and this appears to be a discriminative feature compared to other forms of arthritis such as RA.

### RORγt inhibition selectively blocks innate-like T cells

**Fig. 6:**
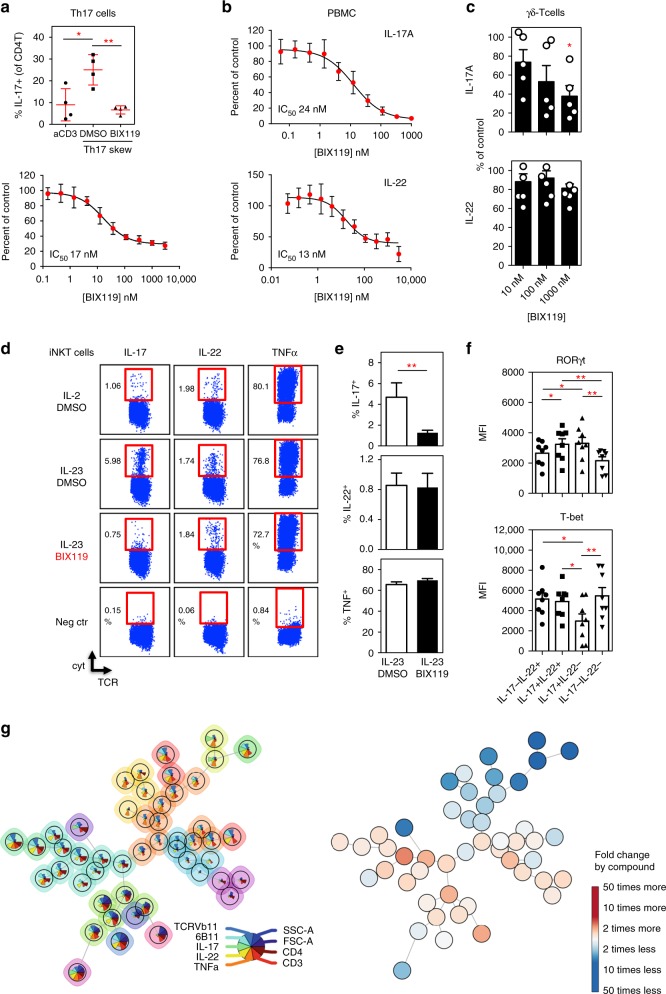
RORC inhibition selectively blocks innate-like T cell function. **a** Upper panel: healthy donor CD4^+^ T cells cultured under Th17 conditions for 7 days. Data is represented as % IL-17^+^ cells as determined by intracellular flow cytometry (*n* = 4). (*p < 0.05, **p < 0.01 as determined by ANOVA). Lower panel: Skewed CD4^+^ Th17 cells were re-stimulated under Th17 conditions for 48 h and IL-17 levels were measured in the supernatants. Dose response of the RORγt antagonist (BIX119) for three donors is shown. **b** Human PBMC activated with aCD3Ab and IL-23 for 72 h. Dose response of the RORγt antagonist is shown for IL-17A and IL-22 secretion detected in culture supernatants by ELISA (*n* = 3). **c** γδ-T cells were activated with anti-CD3/CD28 beads and IL-23 cocktail for four days to produce IL-17A and IL-22 in the presence of increasing concentrations of RORγt antagonist BIX119 (*n* = 5). *p < 0.05 as compared to no compound (ANOVA) (**d**) PBMC were stimulated with αGalCer in the presence of IL-2 or IL-23 cocktail with addition of BIX119 (1 µM) or DMSO. After 14 days, cells were restimulated with PMA/CaI (with BFA) for intracellular cytokine detection. One example representative for three independent experiments is shown. The negative control (neg ctr) consisted of IL-23 skewed cells cultured in BFA alone. **e** quantitative overview of frequency of IL-17, IL-22 and TNFα+ iNKT cells from assays described in D (**p < 0.01 by paired *t*-tests). **f** IL-23 skewed iNKT cells were analyzed for RORγt and T-bet expression by intranuclear flow cytometry. Gating on cytokine producing iNKT cells was performed as illustrated in supplementary figure 7C (*p < 0.05, **p < 0.01 ANOVA). **g** FlowSOM visualization (as explained in Fig. 3F) of IL-23 skewed human iNKT cells (*n* = 3), cultured in the presence or absence of the BIX119 RORγt antagonist (compound). Pie chart shows markers used for automated clustering of iNKT cell types. Data throughout this figure are presented as mean ± SEM

Because Il-17 and/or Il-23 inhibition has demonstrated marked therapeutic efficacy in psoriasis, PsA and AS, a potential alternative strategy therefore includes RORγt inhibition by cost-effective small molecules. However, there are still several unanswered questions as to the impact of RORγt blockade on IL-17 and/or IL-23 mediated immunity as a consequence thereof and whether this impacts all Th17 related cytokines similarly. Several RORγt antagonists or inverse agonists (such as digoxin, SR1001 TMP778, GSK805, and MRL-871, JNJ-54271074) have been identified and tested in different disease models^55–59^. Here, we describe a new and very potent RORγt agonist/inhibitor, BIX119. This compound strongly bound to and selectively inhibited the human RORγ ligand-binding domain. In functional cell assays, BIX119 clearly abolished the induction of Th17 cells from human CD4^+^ T cells and dose dependently suppressed IL-17 by reactivated Th17 cells (Fig. 6a), verifying that it is a selective and potent RORγt inhibitor. It abolished IL-17 and IL-22 production in both CD4^+^ T cell and bulk PBMC cultures upon TCR stimulation (Fig. 6b and Supplementary Fig. 7A). Surprisingly, when BIX119 was added in cultures of IL-23 skewed human iNKT and γδ-T cells, we observed a significant inhibition of IL-17 but not of IL-22 secretion (Fig. 6c-e). Likewise, restimulation of IL-23 skewed cells in the presence of BIX119 led to a selective blocking of IL-17 but not IL-22 secretion (Supplementary Fig. 7B).

Human iNKT cells display a profound heterogeneity with regard to IL-17 and IL-22 production. We found single IL-17 and single IL-22 producing cells, next to a significant subset of double (IL-17+IL-22+) cytokine producers (Supplementary Fig. 7C). Interestingly, IL-17+IL-22− and IL-17+IL-22+cells expressed equally high levels of RORγt whereas single IL-17 producers clearly differed from other subsets by reduced T-bet levels (Fig. 6f). The IL-22 single producers had an intermediate level of RORγt expression (Fig. 6f). We also subjected this dataset to FlowSOM analyses and found that BIX119 significantly inhibited induction of several IL-17 producing iNKT subsets (Fig. 6g and Supplementary Fig. 8). RORγt inhibition shifted the balance from IL-17 producing cells towards a iNKT1 phenotype, while also increasing the prevalence of particular subsets of IL-22 producing cells (Fig. 6g and Supplementary Fig. 8). Thus, RORγt inhibition does not only seems to repress iNKT17 immunity, it bypasses subsets of IL23 induced IL-22 producing cells.

Our data show that innate-like T cells such as iNKT and γδ-T cells are expanded and functionally skewed towards Il-17 secretion in SpA, diseases in which barrier integrity loss in skin or gut seems to sensitize the disease. Importantly, these subsets characterized by a unique transcription factor profile dominated by RORγt are clearly expanded in the circulation and even more in inflamed joints, highlighting the importance of these subsets in SpA. Quite unexpectedly we found that innate-like T cells differ substantially from conventional Th17 cells in cytokine regulation by RORγt^57,60,61^, Earlier in-vivo studies indicated that transient blocking of RORγt allows for selective targeting of Th17 cells without influencing protective ILC3 responses^62^. In addition, pharmacological inhibition of RORγt can modulate TCRα gene rearrangement taken place in developing thymic T lymphocytes and by this moderately limited the T cell repertoire diversity in peripheral circulation^63^. It was not reported whether this influenced iNKT development in treated mice, a finding shown in patients with loss-of-function mutations in the *RORC* gene^64^. By unsupervised computational analysis of flow cytometry data we uncovered a previously unappreciated complexity of both human γδ-T and iNKT cells as we show that RORγt inhibition not only efficiently blocks Th17 function, but also ablated human IL-17 producing γδ-T cells and iNKT cells while selectively sparing IL-22+ subsets. These results confirm that IL-22 can be secreted by innate-like T cells independently from RORC driven IL-17 production, potentially by AHR signaling. Given the established gut-joint axis in SpA, this can be of clinical importance as IL-22 plays a protective role at gut barrier surfaces^10^, sites known to be enriched in innate-like (T) cells. AHR signaling in gut mucosal ILC cells has been shown to be responsible for an IL-22 dependent modulation of immune responses against mucosal microbiota and protecting mice from exacerbated colitis^65,66^. Given that IL-17 also mediated protective barrier function in the gut, these data point to a delicate and complex network of pro-inflammatory and anti-inflammatory signals involved in host protection. A shift in these immune processes in susceptible individuals may tip the balance toward disease initiating events in SpA^67^. Whether AHR mediated responses are altered is appealing given the increased *AHR* gene expression we observed in blood circulating iNKT cells in SpA patients. This however warrants additional investigation of innate-like T cells in gut samples from SpA patients^67^.

A decrease of blood iNKT cells and a relative enrichment of these cells in the joint compartment of SpA patients may also reflect shifts in iNKT cell subsets due to migration of pathogenic subsets toward sites of inflammation. In vivo evidence for such a scenario was very recently provided by the Kronenberg team showing an accumulation of iNKT cells in ankle joints upon progression of mannan induced arthritis in SKG mice^68^. Interestingly, this coincided with a progressive increase of iNKT17 cells in the inflamed joint tissue at the expense of protective IFNγ secreting iNKT1 cells. Alternatively, iNKT1 cells in SpA could be more susceptible towards transition to an iNKT17 cell phenotype under inflammatory conditions^40^. T-bet acts as a repressor of the IL-23/IL-17 immune pathway^69^. So the low T-bet expression in innate-like T cells observed in SpA patients could contribute itself to uncontrolled IL23 mediated pathology.

Collectively these data point to a distinctive regulation of innate-like T cells in SpA with a Th17-skewed phenotype and our data are clearly supportive for the progress in putting forward RORγt antagonism, targeting these cells, as a potential promising new therapeutic approach.

## Methods

### Human samples

Blood samples were obtained from 27 healthy control subjects and 33 patients with newly diagnosed SpA. Synovial fluid (SF) was obtained from patients with an active knee synovitis and an indication for aspiration. Rheumatoid arthritis (RA, *n* = 17) and Crystal-induced arthropathies (CrA, *n* = 11) patients, were included as a chronic and acute inflammatory arthritis comparison group, respectively. For details on the demographic and clinical characteristics of the patients, see Supplementary Table 1. Diagnosis and classification of patients was made by staff rheumatologists following the EULAR/ American College of Rheumatology (ACR) 2010 or ASessment in Ankylosing Spondylitis (ASAS) 2010 criteria. Patients were treatment naïve or under treatment with a NSAID and/or DMARD (Methotrexate, Sulfasalazin), but naïve for treatment with biologicals at the time op sampling (see Supplementary Table 1). Ultrasound guided synovial biopsy sampling of knee joints was done by an experienced rheumatologist in our early arthritis clinic. At least six biopsies were taken to ensure enough cellular material (>500,000 cells) could be extracted for flow analyses. This study was approved by the local ethics committee of Ghent University Hospital and all patients and healthy controls gave written informed consent.

### Cell isolation

PBMC and SFMC were directly isolated from PB and SF samples as described before^70^. Synovial tissue samples were digested with a mixture of Collagenase IV and VIII (Sigma, both 1 mg/ml) for 30 min at 30 °C under continuous stirring conditions. Digested samples were filtrated (40 µm), washed and prepared for flow cytometric analyses as indicated below. Blood γδ-T cells (TCRγδ+) and iNKT cells (TCRVα24+ TCRVβ11+) were sorted on a FACSAria III (BD). The latter cells were expanded by 6B11 stimulation as described in ref. ^71^. IL-17+ iNKT cells were isolated by means of an adapted IL-17 Capture Assay (Cell Enrichment and Detection Kit, Miltenyi Biotec). Briefly, two weeks after αGalCer (100 ng/ml) and IL-23 stimulation, PBMC were restimulated with Cytostim (Miltenyi Biotec; crosslinking TCR and MHC) for 4 h at 37 °C. Subsequently, cells were labeled with IL-17 Catch Reagent, by which cell secreted IL-17 is bound at the cell membrane. Finally, cells are labeled with an APC labeled IL-17 detection antibody, next to 7-aminoactinomycin D (7-AAD; for life/dead discrimination) and antibodies directed against TCRVα24 and TCRVβ11, prior to FACSorting of IL-17^+^ TCRvα24TCRvβ11^+^ 7-AAD^neg^ cells. A negative (no Cytostim) and positive (PMA/CaI/BFA) control condition was used in each experiment.

### Cell cultures

For iNKT assays, PBMC were cultured in 24-well plates (1.5 × 10^6^ cells/well) for 14days in the presence of αGalCer (100 ng/mL, in house made) and IL-23 (20 ng/mL), IL-1β (10 ng/mL), TGFβ1 (5 ng/mL; all eBioscience), and IL-2 (5 U/mL, Roche) or IL-2 alone. Similar experiments were performed with sorted iNKT and γδ-T cells in U bottom 96 well plates (50,000 cells/well) where we added 50,000 irradiated (40 Gy) T cell depleted PBMC cells (using CD2 dynabeads, ThermoFisher) as feeder/antigen presenting cells. Cytokine titers in supernatants were then determined by ELISA (ebioscience) or multiplex protein assays (MSD).

### SFMC depletion assay

SFMC from SpA patients (*n* = 3) were split in two equal fractions (I and II). Both fractions underwent a similar sorting procedure to exclude a potential bias on functionality. Fraction I was labeled only with 7-AAD to exclude dead cells. Cells from fraction II were labeled with anti- human TCRVβ11, 6B11 and TCRγδ and 7-AAD. Fraction I and II were acquired on a FACSAria III sorter (BD) at the same flow rate and gated for live lymphocytes (7-AADneg FSC^lo^/SSC^lo^). Fraction II was depleted of iNKT (TCRVβ11+ 6B11+) and γδ-T cells (TCRγδ+) cells by negative gating (see Fig. 4c). Equal cell numbers (min. 300,000) from sorted SFMC and SFMC cells depleted of iNKT and TCRγδ cells (ΔSFMC) were cultured in 96 flat bottom plates. For TCR stimulation, wells were (prior to cell adition) plate coated with aCD3Ab and aCD28Ab (both at 5 µg/ml in PBS, BD) overnight at 4 °C. Wells indicated for non-TCR stimulated conditions were incubated with PBS only. Wells were washed extensively before cells were added. IL-23 (20 ng/ml) together with IL-1β (10 ng/ml) and TGFβ1 (5 ng/ml) resuspended in culture medium was added to indicated wells (“IL-23” condition). Non cytokine stimulated wells were incubated with medium only (“no IL-23”). Supernatants of cultures were collected at 72 h of culture and in levels of IL-17, IL-22, TNFα, and IFNγ were determined by ELISA (eBiosciences).

### Flow cytometry

Eight–ten color flow cytometry was applied to PBMC and SFMC samples and isolated (cultured) cells as described before^70,71^, using fluorochrome conjugated antibodies as summarized in Supplementary Table 2. iNKT cells (CD3+ 6B11+ TCRVβ11+), γδ-T cells (CD3+ TCRγδ+) and CD161+ and CD161− conventional Tconv (T cells excluded from innate-like T cells, CD3+ TCRγδ− TCRVα7.2-TCRVα24Jα18−) were gated as shown in Supplementary Fig. 1A. Human aGalCer loaded CD1d tetramer (in house produced) was used to verify iNKT stainings as indicated in Supplementary Figure 1E-F). FcR Blocking Reagent (Miltenyi Biotec) was used to exclude aspecific FcR binding of Abs. For flowcytometric analyses of cultured cells, Ab staining was preceded by incubation with viable cell discriminating reagents (LIVE/DEAD® Fixable Violet Dead Cell Stain Kit, ThermoFisher or Fixable Viability Stain 520, BD). A minimum of 500 iNKT and 3000 γδ-T cells were acquired on a FACS Canto II or Aria III machine (BD) to guarantee accurate calculations of the expression of differentiation markers/transcription factors by these cells. Data analyses and graph presentations were done by means of FlowJo software, v9.8 and 10.2 (Treestar). Prior to intracellular staining of transcription factors, the cells were fixed and permeabilized using the reagents and following the instructions of the FoxP3 intracellular staining kit (eBioscience). For cytokine stainings, cells were collected and restimulated for 4 h with PMA (25 ng/mL), calcium-ionomycin (1 µg/mL; both from Sigma) and brefeldin A (10 ng/mL; BD). Subsequently, cells were processed using the Cytofix/Cytoperm kit (BD) as described before^70^.

*RORC* and *Il23R* mRNA expression on specific T cell subsets was measured by means of a PrimeFlow RNA Assay (eBioscience), using VA1 and VA4 probes, respectively, and following the manufacturer’s instructions. Proper positive and negative controls (RPL13A and FMO) were included in each assay.

### qPCR

Cells were lysed using RLT lysis buffer supplemented with βmercapto-ethanol (Qiagen) and were kept at −80 °C until analysis. RNA extraction and cDNA preparation were conducted using the RNeasy mini kit (Qiagen) and the QuantiTect Reverse Transcription Kit (Qiagen), according to the manufacturer’s instructions. qPCR reactions were performed in duplicate using a LightCycler 480 system (Roche). QuantiTect primer assays for *IL17A, IL17F, IL22, RORC, and IL23R* genes were ordered from Qiagen. Reference (housekeeping) genes were designed, validated and tested for stable expression (including Gapdh, β-actin and HPRT) and data were analyzed using qbase+ (Biogazelle).

### RNASeq

PBMC were isolated from freshly taken blood samples of treatment naïve SpA patients (*n* = 7) and RA patients (*n* = 5) as described above. Cells were labeled with anti-human TCRVβ11, 6B11, CD161, CD3, TCRγδ and 7-AAD to exclude dead cells. iNKT (CD3 + TCRVb11 + 6B11+), γδ-T cells (CD3 + TCRγδ+) cells and Tconv (CD3 + CD161-; negative for iNKT and γδ-T markers) were sorted on a FACSAria III (BD) and immediately stored in RLT (Qiagen) +1% β-Mercaptoethanol (Sigma) at −80 °C. RNA was isolated by means of the RNeasy Micro kit following manufacturer’s instructions (Qiagen). RNA concentration and purity were determined spectrophotometrically using the Nanodrop ND-1000 (Nanodrop Technologies) and RNA integrity was assessed using a Bioanalyser 2100 (Agilent). Per sample, an amount of 100 pg (iNKT) or 1 ng (γδ and Tconv) of total RNA was used as input for the SMART-Seq v4 Ultra Low Input RNA protocol (version 091817) from Takara Bio USA, Inc. Subsequently, 1 ng (iNKT) or 2 ng (γδ-T and Tconv samples) of purified cDNA was sheared to 300 bp using the Covaris M220. From the sheared material, sequencing libraries were prepared with the NEBNext Ultra DNA Library Prep Kit for Illumina (version 6.0–2/18), according to the manufacturer’s protocol including a size selection to 250 bp insert size. Sequence-libraries of each sample were finally equimolarly pooled and sequenced on 4 NextSeq500 v2 flow-cell at 1 × 75 bp (76–8–8–0). The preprocessing of the RNA sequencing data was done by Trimmomatic. The adapters were cut off, and reads were trimmed when the quality dropped below 20. Reads with a length <35 were discarded. All samples passed quality control based on the results of FastQC. Reads were mapped to the human reference genome (hg19) via Tophat2 and counted via HTSeqCount. Samples were subsequently analyzed using R/Bioconductor, and the R package limma was used to normalize the data and to do differential expression analysis. The 3 cell types were analyzed separately and we corrected for batch effects using ComBat of the R package sva. For the analysis of differentially expressed (DE) genes, we applied a stringency level where the adjusted p-value was less than 0.05 and the log2FC was less than −1 or greater than 1. For the heatmap we scaled the log2 expression values per gene by calculating the mean expression per gene and then substracting that mean value of each expression value.

### RORγt compound

The RORγt agonist/inhibitor, BIX119, was discovered through screening a small-molecule compound library. BIX119 strongly bound to the human RORγ ligand-binding domain (LBD) and was active in a RORγ LBD reporter assay (Kd for RORγ LBD – 65 nM; IC50 for RORγ LBD reporter assay 260 nM). The compound showed high selectivity towards RORγt as demonstrated by a lack of significant activity against RORα (IC50 > 10 μM) and RORβ (IC50 > than 6 μM). In addition, BIX119 was inactive against a panel of 25 nuclear receptors up to 10 μM. BIX119 potently inhibited IL-17A secretion from human PBMCs activated with anti-CD3Abs and IL-23, as well as CD4^+^ Th17 cells stimulated with anti-CD3/CD28/CD2 beads and a Th17 cytokine cocktail including IL-1β, IL-23, IL-6, and TGFβ (IC_50_ 10 nM and 17 nM, respectively). Levels of IL-17 and IL-22 in supernatants of compound experiments were determined by means of ELISA using respecitively the human IL-17A Tissue Culture Kit (Meso Scale Discovery) and the Human IL-22 Quantikine Kit (R&D Systems).

### FlowSOM

Multiparameter flow cytometry data derived from patient and control samples, were subjected to FlowSOM^21^, which uses a self-organizing map (SOM) to cluster and visualize the selected cell subsets (iNKT, TCRγδ, and Tconv) in different nodes based on the expression of distinct markers included in the flow Ab panel. For each subset, an aggregated file was created containing the cells from the different samples, compensated and transformed as specified by the FlowJo workspace and for the resulting aggregated files, a FlowSOM tree was trained. A grid of 7 by 7 nodes, resulting in 49 clusters was used, which are visualized using the Minimal Spanning Tree visualization. Scaling was set to FALSE and the number of metaclusters to 10, all other parameters were kept as default. The legends in the figures indicate which markers were used for clustering. The individual files were mapped onto the trees to compute the percentage of cells assigned to each cluster. The mean percentage per sample group was determined, and the log10 fold change was computed to visualize the differences between the groups. The log10 fold change was manually set to −2 (or a decrease by a 100 times less) if no cells where present in the patient group (corresponding with a theoretical fold change of -infinity). A similar strategy was applied for the compound data, with log10 fold change calculated for the condition with compound vs. no compound (only vehicle). Additional information related to the interpretation of the FlowSOM tree figures is included in the figure legends.

### Statistics

Data were analyzed using Prism software v5.0 (GraphPad) and SPSS version 23 software. The Shapiro-Wilk test was used to test normality of the data. Statistic tests (and annotations) are mentioned in the figures and figure legends. An overview of statistics for Fig. 1f is provided in the Supplementary Table 3. In general, the two-sided non-parametric Mann–Whitney *U* test was performed for comparison of non-normality numeric data, whereas two-sided student’s (paired) *t* test were used for normally distributed data. One-way analysis of variance (ANOVA) were used for multi-group analyses of normally distributed data. Data is presented as mean ± SEM unless stated otherwise. All the statistical assessments were made at the 95% confidence interval; *p* < 0.05 was considered to be statistically significant.

### Reporting Summary

Further information on experimental design is available in the Nature Research Reporting Summary linked to this article.

## Supplementary information

Supplementary Information

Reporting Summary

## Data Availability

RNA-seq data from this study is deposited in GEO with accession code GSE122624. Other data that support the findings of this study are available from the corresponding authors upon reasonable request.
