# Supplementary material for: RORγt inhibition selectively targets IL-17 producing iNKT and γδ-T cells enriched in Spondyloarthritis patients
Source: Nat Commun. 2019 Jan 2;10:9. doi: 10.1038/s41467-018-07911-6 (PMC6315029; doi:10.1038/s41467-018-07911-6)
Supplement: Supplementary file 1 — Supplementary Information [file 41467_2018_7911_MOESM1_ESM.pdf]

SUPPLEMENTARY INFORMATION

Venken et al.

RORγt inhibition selectively targets IL-17 producing iNKT and γδ-T cells enriched in Spondyloarthritis patients

Supplementary Figure 1. Identification of blood circulating RORγt + iNKT and γδ-T cells

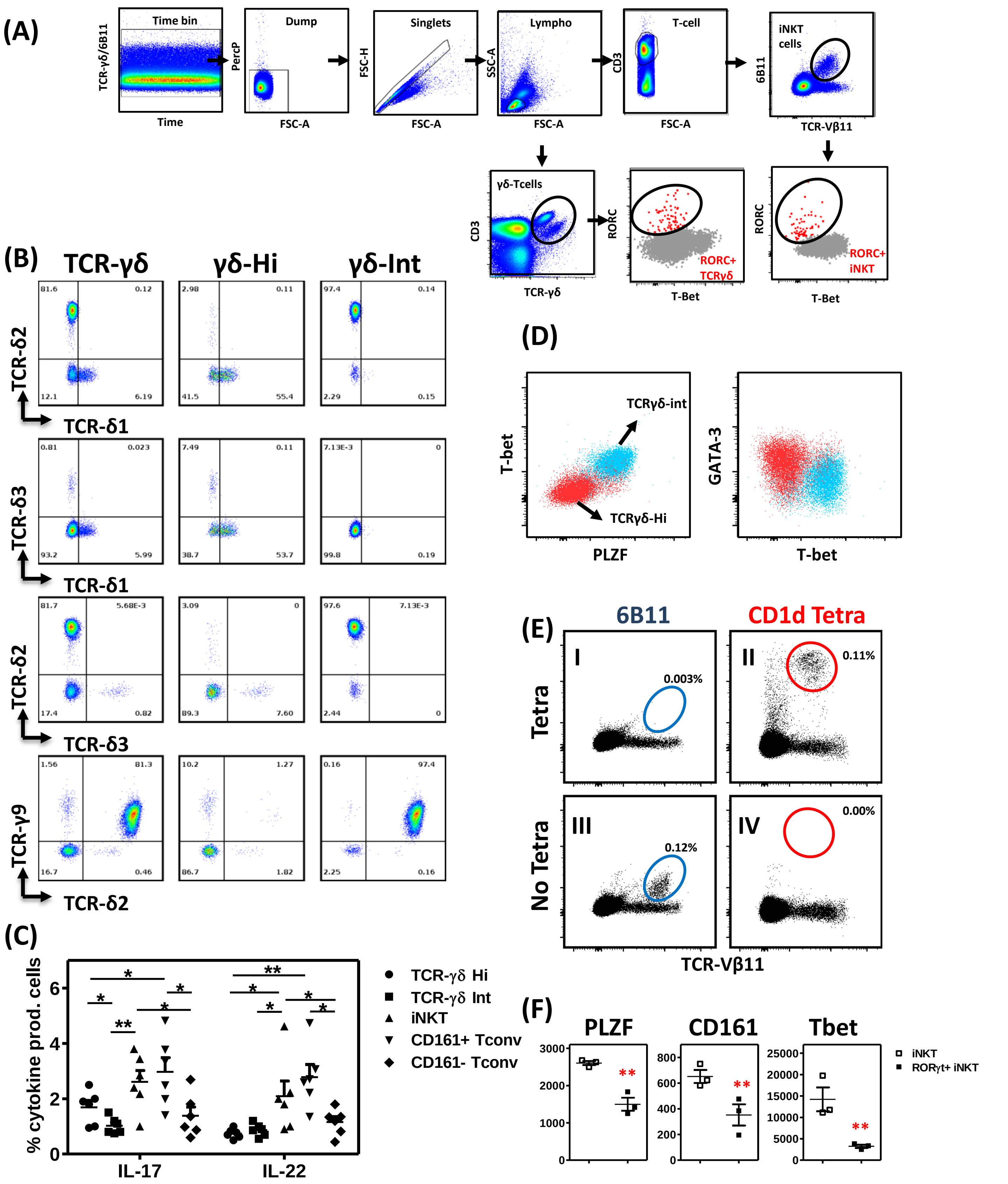

(A) Gating strategy for identification of RORγt⁺T-bet^{lo} iNKT and γδ-T cells. Proper gates were drawn for exclusion of debris, doublets, non-viable cells. A time bin was used for quality control of fluorescent data (erroneous fluctuations in fluorescent signals) and to exclude machine malfunctioning. A dump channel was applied to take into account autofluorescence of cells. **(B)** γδ chain profiling (by flow cytometry) of indicated γδ-T cells. One experiment is shown, representative for n=3. Upper three rows of plots show different δ chain expression profiles (as shown in different combinations). Lower row shows TCRVγ9δ2 expression of subsets. **(C)** quantitative data (related to Figure 1D) showing IL-17 and IL-22 production by T cell subsets (*p<0.05, ** p<0.01 ANOVA). **(D)** Overlaid costaining of T-bet and PLZF/GATA-3 of TCRγδ-Hi (red) and TCRγδ-int (blue) cells. **(E)** PBMC cells (n=3) were pre-stained with (I-II) or without (III-IV) aGalCer-CD1d Tetramer and subsequently incubated with 6B11 and TCRVb11 Abs. Prestaining with Tetramer blocked 97-99% of 6B11 staining indicating that majority of Tetra+ cells and 6B11+ cells are identical. One representative example is shown. **(F)** CD1d Tetramer+ cells were fixed and intracellularly stained for transcription factors (and CD161). Data expressed as MFI of indicated markers for Tetra+ cells or Tetra+ RORγt+ cells (comparable results for 6B11+ cells). (** p<0.01 paired t-tests). Data throughout this figure are presented as mean±SEM.

Supplementary Figure 2. IL-23R and RORC expression by iNKT and γδ-T cells

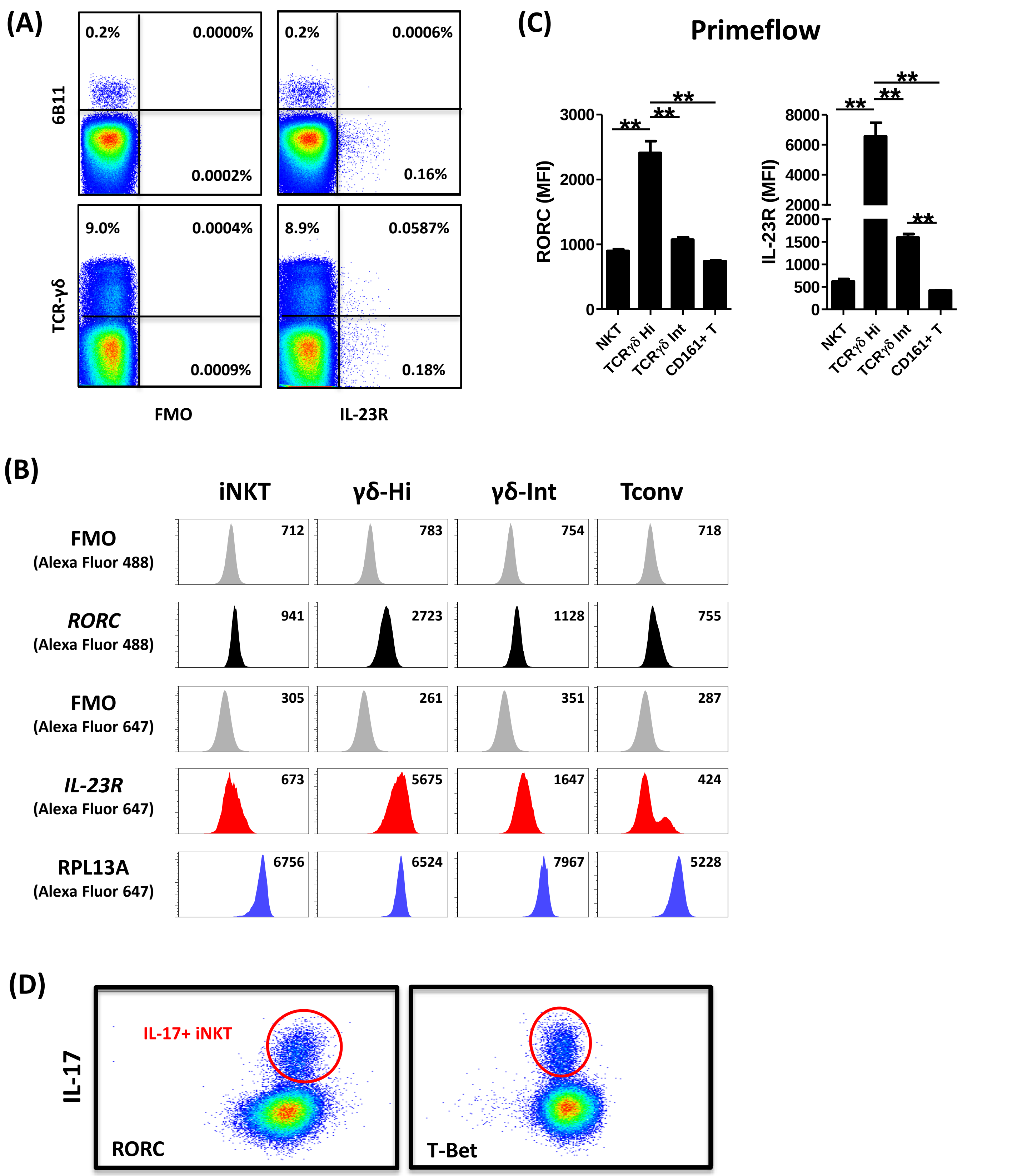

(A) IL-23R expression measured on iNKT cells (6B11+) and γδ-T cells (TCRγδ+) present in PBMC of a healthy control. Quadrant axes were set based on FMO staining. **(B)** *RORC* and *Il23R* mRNA expression on specific T cell subsets (in PBMC) was measured by means of a PrimeFlow RNA Assay (eBioscience), using respectively VA1 and VA4 probes, and following the manufacturer's instructions. Type 1 human IL23R Alexa Fluor® 647 Target Probe Set and Type 4 human RORC Alexa Fluor® 488 Target Probe Set were used for the detection of respectively human IL23R and RORC mRNA in indicated cell types. FMO represent cells stained under similar staining conditions (including fluorescent label probes) with the exclusion of target probes. Probes for human ribosomal protein, RPL13A (Type 1 Human RPL13A Alexa Fluor647), were used as a positive control. Numbers in histograms represent MFI. **(C)** Quantitative data from PrimeFlow assays depicted in Figure 2A. Bars represent mean expression values +/- SEM (n=3). (*p<0.05, ** p<0.01 ANOVA). **(D)** PBMC were cultured with αGalCer in the presence of IL-23, IL1b, TGFb1 to induce IL-17 cytokine response. IL-17 expression was plotted against RORC or Tbet expression of gated iNKT cells.

Supplementary Figure 3. iNKT and γδ-T cells in SpA synovial tissue

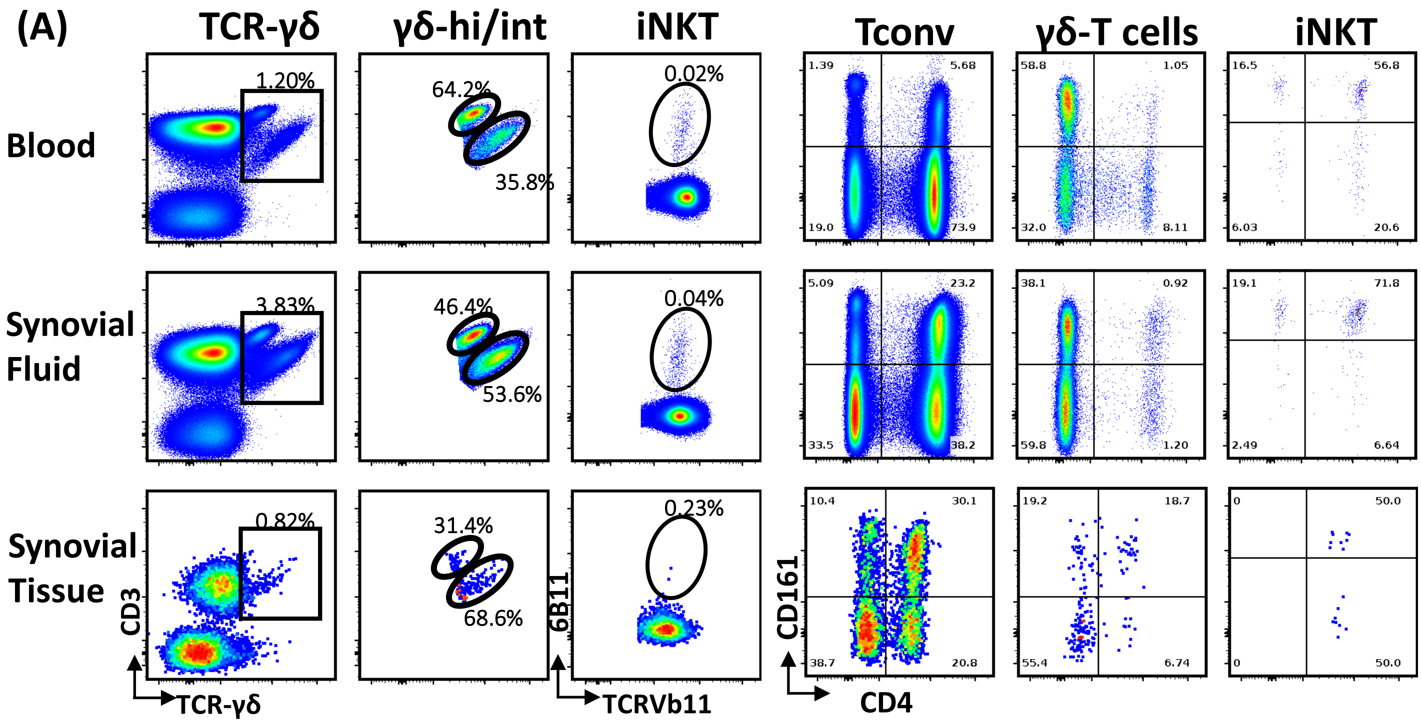

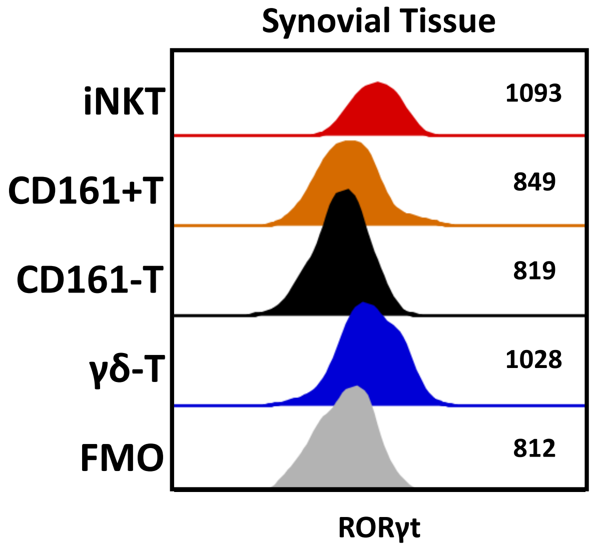

Upper panel: Paired blood (PBMC), synovial fluid (SFMC) and synovial tissue (ST) samples showing enrichment of iNKT and TCRγδ-Hi subsets in inflamed joints (with indicated CD4CD161 expression phenotype). One example shown, representative for n=2. In general, higher percentages of IL-17+ cells were observed in CD4+ T cell subsets as compared to their CD4- counterparts (our unpublished data). CD4+ Tconv and CD4+ γδ-T cells showed a relative increase of CD161+ cells in the joint compartment (Tconv: 7.1%, 37.8% and 57.1% and γδ: 11.5%, 43.4% and 73.8% CD161+ for respectively PB, SF, and ST cells of this patient) whereas synovial CD4+ iNKT cells were enriched for CD161 negative cells in the joint tissue (73.4, 91.5 and 50% CD161+ for respectively PB, SF, and ST cells). **Lower panel**: RORγt expression on indicated T cell subset present in patient derived synovial tissue. One example shown representative for n=2. Indicated values represent MFI.

Supplementary Figure 4. FlowSOM analyses of innate-like and conventional T cells of SpA patients

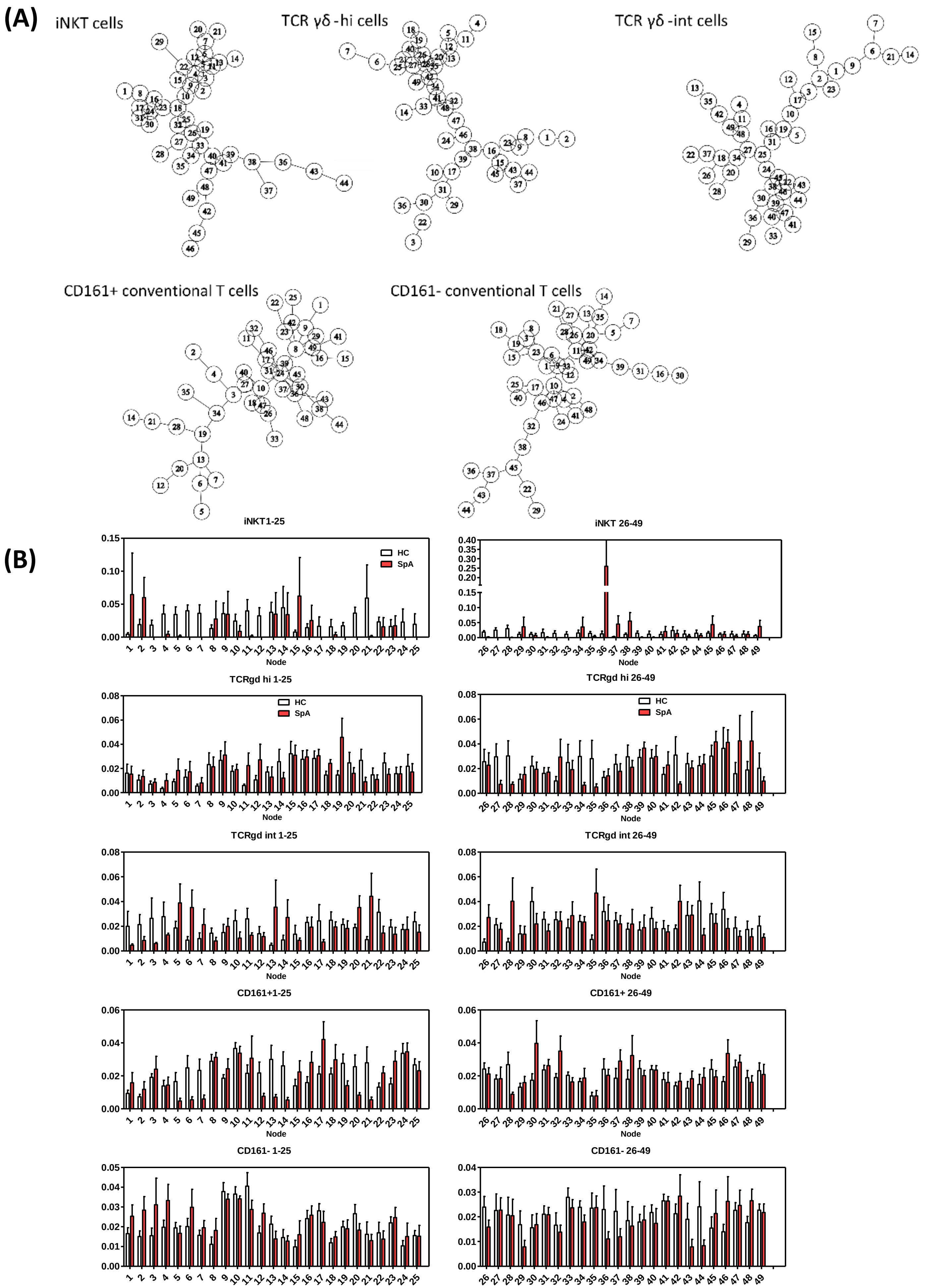

Supplementary FlowSOM graphs related to Figure 3F showing numbering of nodes in FlowSOM trees (A) and showing expression levels of T cell subsets in Spa vs. HC (B). Data throughout this figure is presented as mean±SEM.

Supplementary Figure 5. FlowSOM analyses of γδ-int T cells and conventional T cells subsets in SpA versus healthy individuals

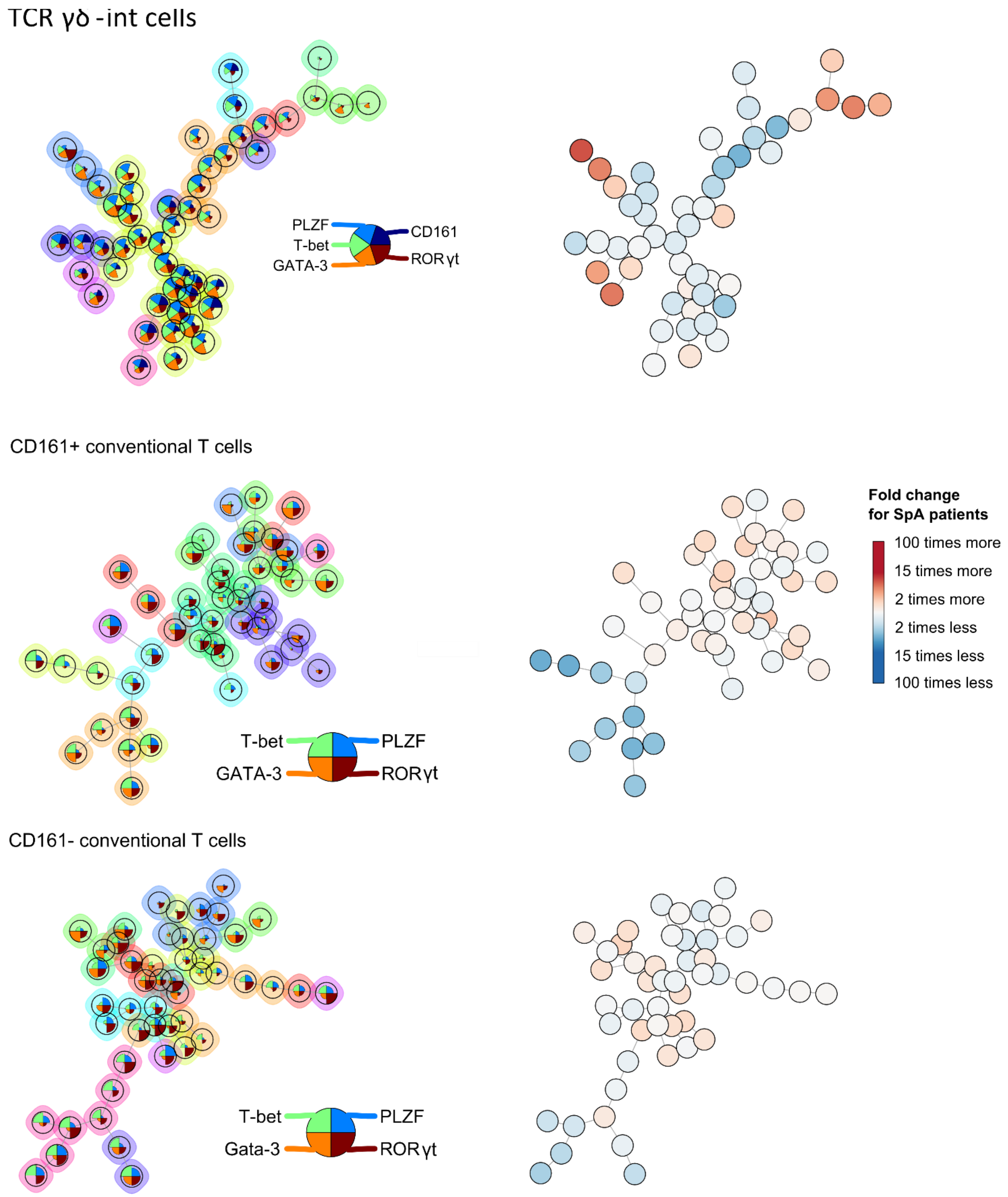

FlowSOM tree analyses of γδ-int and Tconv cells (see related Fig. 3F for further explanation).

Supplementary Figure 6. IL-17 production by SpA innate-like Tcells and conventional T cells

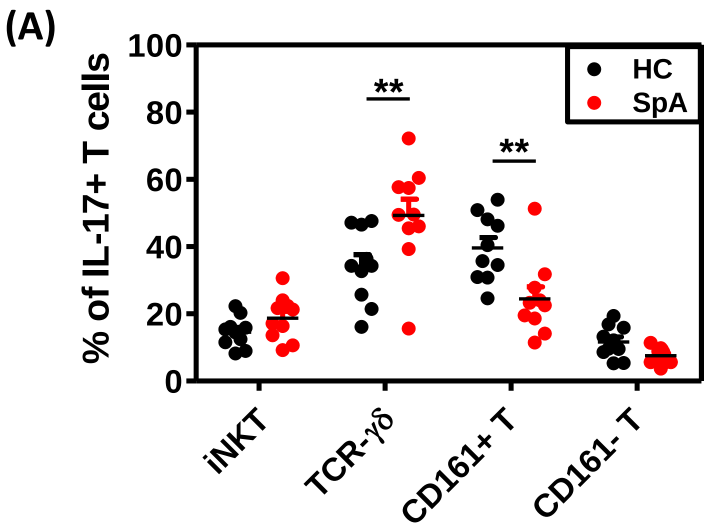

(B) SFMC depletion experiments

PHA stimulation

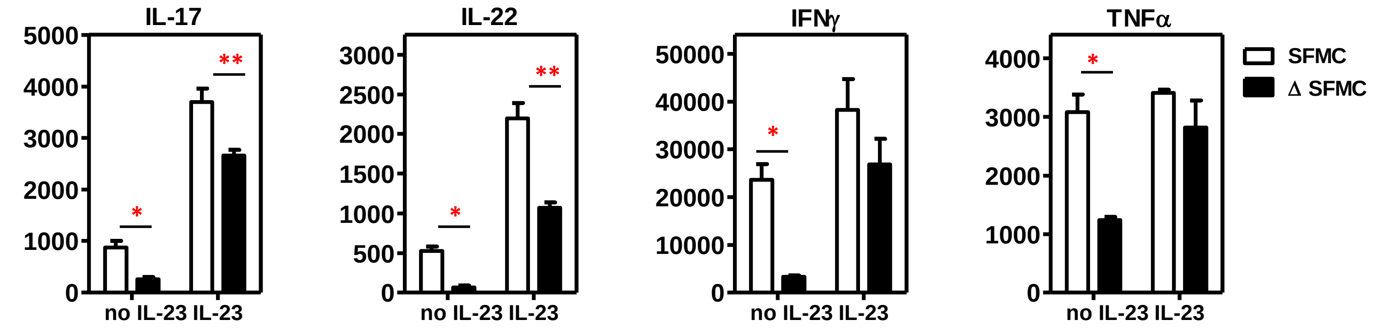

IL-23 skew and PMA/CaI restimulation

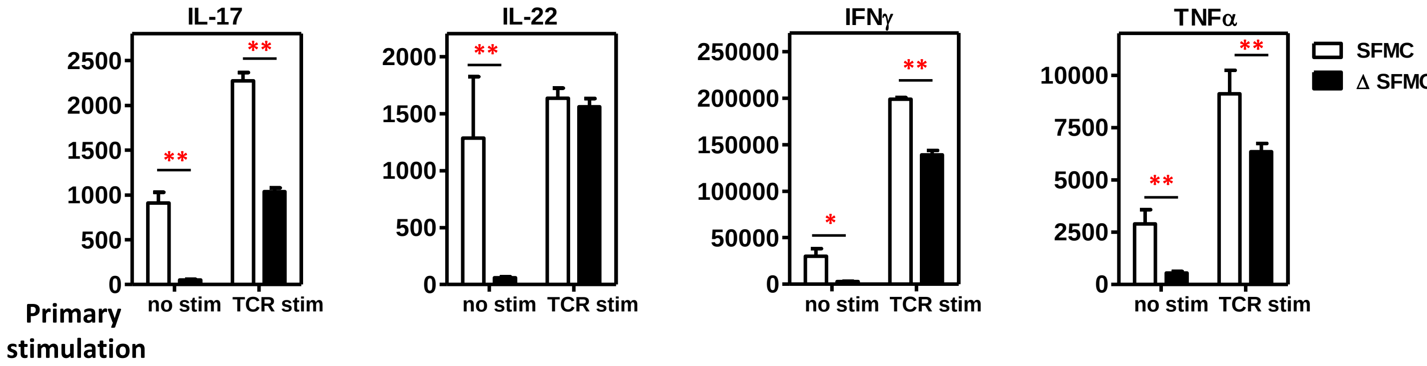

(A) IL-17 production by iNKT, γδ-T cells, Tconv cells from SpA patients (n=10) and controls (n=10) as measured by intracellular cytokine staining of PBMC after 4h incubation of cells with PMA/CaI in the presence of BFA. Relative representation of indicated T cell subsets among IL-17 producing T cells for each individual. Mean number (+/- SD) of IL-17+ Tcells: 1094 +/- 622 and 1693 +/- 1077 for respectively HC and SpA patients. ** p<0.01 ANOVA. **(B)** SpA derived synovial fluid derived mononuclear cells (SFMC) and SFMC cells depleted of iNKT and TCRγδ cells (ΔSFMC) were stimulated with PHA for 72h in the presence or absence of IL-23 and supernatants collected (upper panel). In a second set-up, the SFMC and ΔSFMC cells were skewed with IL-23 in combination with aCD3/aCD28Ab (TCR stim) or without TCR stimulation (no stim). At day 7, cells were stimulated with PMA/CaI for 24h and supernatants were collected of these cultures (lower panel). Cytokine secretion was measured by ELISA. SFMC vs ΔSFMC; * p<0.05, ** p<0.01 two-way ANOVA. Results shown from one experiment with triplicate wells (representative for n=2). Differences with regard to the magnitude of the cytokine response (with or without cell depletion) in these experiments as compared to Figure 4C (short-term IL-23 and/or TCR stimulation) could potentially be explained by (a) the broader mitogenic effect of PHA, which next to Tcells also stimulate for example (IL-17 producing) ILC cells included in SFMC samples and (b) differentiation of conventional T cells towards the Th17/22 lineage upon IL-23 skewing in the second set-up.

Supplementary Figure 7. RORC inhibition of Th17 and innate-like T cells

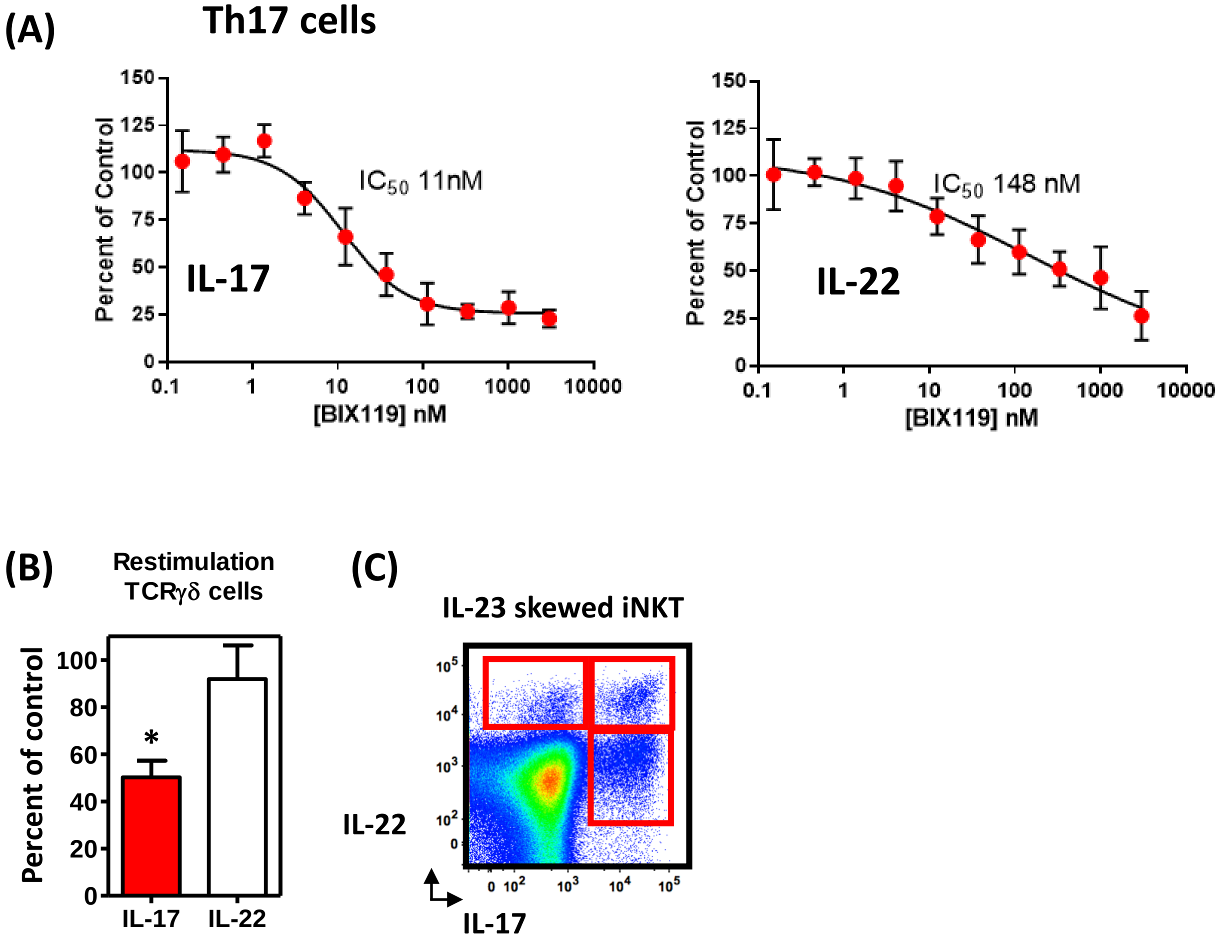

(A) CD4+ T cells stimulated for 72 hours with anti-CD3/28/2 beads in the presence of RORC compound (BIX119) at different concentration. IL-17 and IL-22 levels in the supernatants were measured by means of ELISA and data for each dose was plotted relative to the condition without compound. **(B)** Isolated γδ-T cells were skewed with an IL-23 cocktail and anti-CD3/CD28 beads for four days (with no compound) and then cells were then rested and re-stimulated for 3 days in the absence or presence of RORγt antagonist BIX119. IL-17/IL-22 was measured in supernatants of cultures and data are expressed as mean (+/- SEM) inhibition on IL-17 or IL-22 secretion by addition of BIX119. **(C)** Flow plots showing IL-17A-, IL-22- single along double cytokine producing IL-23 skewed iNKT cells.

Supplementary Figure 8. FlowSOM analyses of RORC inhibited iNKT cells

(A)

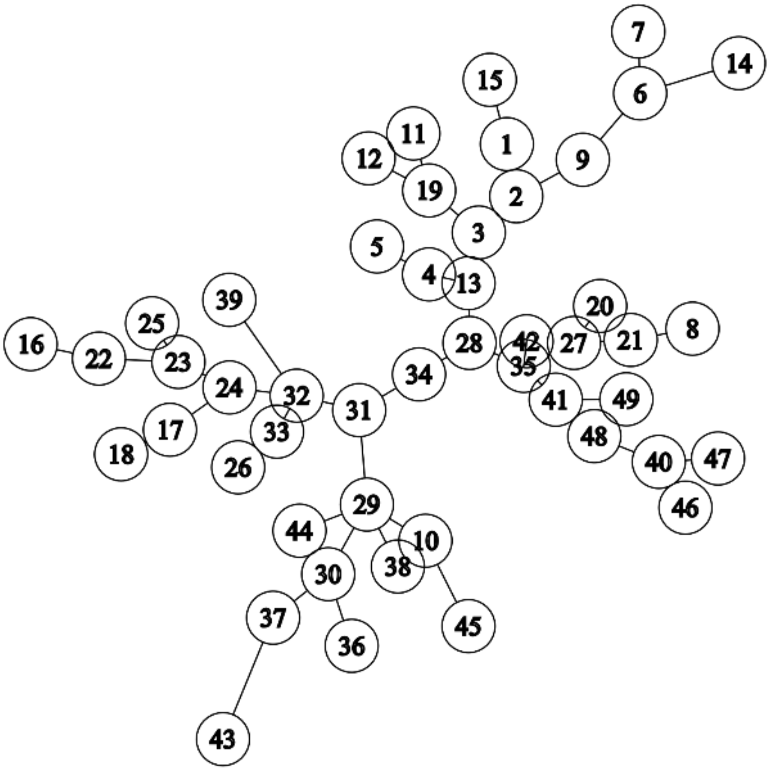

(B)

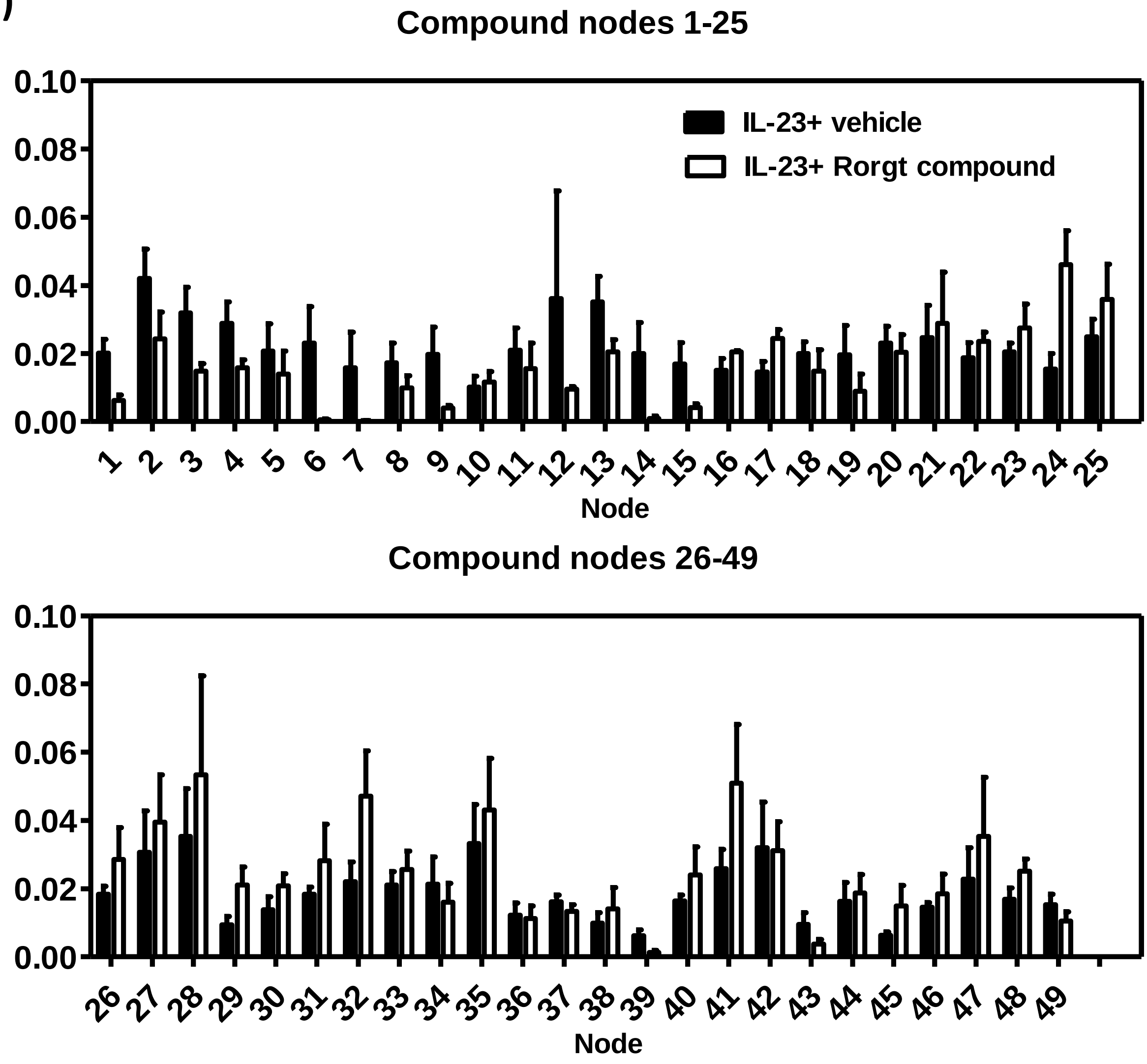

(A-B) Supplementary FlowSOM graphs related to Fig. 6G. Data throughout this figure is presented as mean±SEM.

Supplementary Table 1. Patient characteristics

STUDY POPULATION		SpA	RA	CrA	HC
Number (n)		33	17	11	27
Gender (M/F)		18/15	10/7	6/5	12/15
Age (years)		41.0 ± 12.9	58.2 ± 15.0	65.7 ± 8.7	39.9 ± 14.7
Disease duration (years)		5.2 ± 4.2	5.3 ± 4.4	7.6 ± 5.2	
Swollen Joint count		2.6 ± 2.1	9.3 ± 8.5	2.4 ± 2.2	
CRP (mg/dl)		5.2 ± 3.9	6.5 ± 4.1	9.6 ± 7.8	
ESR (mm/h)		42.3 ± 32.8	45.5 ± 26.2	73.3 ± 27.6	
Treatment	NSAID	20	3	1	
	Corticoid	0	1	0	
	DMARD	10	7	0	
	Biol	0	0	0	

The SpA group consisted of 22 peripheral SpA and 11 axial SpA patients defined according to the new ASAS criteria. CrA group included patients with an acute form of gout and chondrocalcinosis. Abreviations: Spondyloarthritis (SpA), rheumatoid arthritis (RA), crystal induced arthritis (CrA), healthy control (HC), non steroid anti-inflammatory drug (NSAID), disease modifying antirheumatic drugs (DMARD), biological (Biol).

Supplementary Table 2. Overview of mAbs used in flow cytometry experiments

Target	Ab Clone	Conjugate (fluorescent Tag)	Dilution	Company	Catalogue #
TCRVa24Ja18	6B11	PE	1/100	eBiosciences	12-5806-42
TCRVa24Ja18	6B11	BV510	1/50	BD	563267
TCRVb11	C21	FITC	1/67	Beckman Coulter	IM1586
TCRVb11	C21	PE	1/67	Beckman Coulter	IM2290
TCRgd	B1	BV421	1/67	BD	562560
TCRgd	B1	PercP-efluor 710	1/50	eBiosciences	46-9959-42
CD3	UCHT1	APC-eFluor780	1/100	eBiosciences	47-0038-42
CD161	DX12	APC	1/40	BD	561729
CD161	DX12	BV510	1/50	BD	563212
CD161	191-B8	PE-Vio770	1/50	Miltenyi	130-099-965
CD19	HIB19	PerCP-Cy5,5	1/100	eBiosciences	45-0199-42
CD14	61D3	PerCP-Cy5,5	1/100	eBiosciences	45-0149-42
IL-23R	Clone # 218213	APC	1/20	R&D	FAB14001A-025
CCR6 (CD196)	R6H1	PE-Cy7	1/50	eBiosciences	25-1969-42
TCRVd1	REA173	FITC	1/100	Miltenyi	130-118-362
TCRVd1	REA173	VioGreen	1/100	Miltenyi	130-100-563
TCRVd2	REA771	APC	1/100	Miltenyi	130-111-011
TCRVd3	P11.5B	FITC	1/100	Beckman Coulter	NA
TCRVγ9	REA470	PE	1/100	Miltenyi	130-107-434
CD4	SK3	BV510	1/100	BD	562970
CD8a	RPA-T8	Alexa Fluor 700	1/100	eBiosciences	56-0088-42
CD8a	RPA-T8	APC	1/100	eBiosciences	17-0088-42
RORgt	Q21-559	BV421	1/50	BD	563282
T-bet	O4-46	APC	1/33	BD	561267
T-bet	O4-46	BV421	1/33	BD	563318
Gata-3	TWAJ	PE	1/33	eBiosciences	12-9966-42
Gata-3	TWAJ	PE-Cy7	1/33	eBiosciences	25-9966-42
PLZF	Mags.21F7	Alexa Fluor 488	1/33	eBiosciences	53-9320
IL-17A	eBio64DEC17	PerCP-Cy5.5	1/100	eBiosciences	45-7179-42
IL-17A	eBio64DEC17	APC	1/100	eBiosciences	17-7179-42
IL-17A	SCPL1362	PE	1/100	BD	560436
IL-22	22URTI	PE-Cy7	1/50	eBiosciences	25-7229-42
IL-21	eBio3A3-N2	PE	1/50	eBiosciences	50-7219-42
TNFa	MAb11	PE-Cy7	1/200	eBiosciences	25-7349-82
IFNg	45.B3	PerCP-Cy5.5	1/100	eBiosciences	45-7319-42

Supplementary Table 3. Statistics related to Figure 1F.

Statistics Figure 1F

ANOVA (Bonferroni's Multiple Comparison Test)	**PLZF**		**T-bet**		**GATA-3**		**CD161**	
	Significantly different	Summary	Significantly different	Summary	Significantly different	Summary	Significantly different	Summary
**TCRgd HI vs TCRgd Int**	Yes	***	Yes	**	Yes	***	No	ns
**TCRgd HI vs CD161+ Tconv**	No	ns	No	ns	Yes	***	No	ns
**TCRgd HI vs CD161- Tconv**	No	ns	Yes	**	Yes	***	No	ns
**TCRgd HI vs iNKT**	Yes	***	No	ns	Yes	***	Yes	*
**TCRgd HI vs Rorgt+ iNKT**	No	ns	Yes	***	Yes	***	No	ns
**TCRgd INT vs CD161+ Tconv**	Yes	***	Yes	***	No	ns	No	ns
**TCRgd INT vs CD161- Tconv**	Yes	***	Yes	***	No	ns	Yes	*
**TCRgd INT vs iNKT**	No	ns	Yes	**	Yes	*	No	ns
**TCRgd INT vs Rorgt+ iNKT**	Yes	***	Yes	***	No	ns	No	ns
**CD161+ Tconv vs CD161- Tconv**	No	ns	No	ns	No	ns	Yes	*
**CD161+ Tconv vs iNKT**	Yes	***	No	ns	No	ns	Yes	***
**CD161+ Tconv vs Rorgt+ iNKT**	No	ns	No	ns	No	ns	No	ns
**CD161- Tconv vs iNKT**	Yes	***	Yes	*	No	ns	Yes	***
**CD161- Tconv vs Rorgt+ iNKT**	No	ns	No	ns	No	ns	No	ns
**iNKT vs Rorgt+ iNKT**	Yes	***	Yes	***	Yes	*	Yes	***

yellow: comparison between two subsets of the same T cell population — ***p<0.05**

Blue: comparison between TCRgd Hi vs CD161+ Tconv vs. Rorgt iNKT (all combinations) — **** p<0.01**

green: significant differences — ***** p<0.001**
