# Supplementary material for: RORγt inhibition selectively targets IL-17 producing iNKT and γδ-T cells enriched in Spondyloarthritis patients
Source: Nat Commun. 2019 Jan 2;10:9. doi: 10.1038/s41467-018-07911-6 (PMC6315029; doi:10.1038/s41467-018-07911-6)
Supplement: Supplementary file 2 — Reporting Summary [file 41467_2018_7911_MOESM2_ESM.pdf]

nature research

Corresponding author(s): Elewaut D and Venken K

Reporting Summary

Nature Research wishes to improve the reproducibility of the work that we publish. This form provides structure for consistency and transparency in reporting. For further information on Nature Research policies, see Authors & Referees and the Editorial Policy Checklist.

Statistical parameters

When statistical analyses are reported, confirm that the following items are present in the relevant location (e.g. figure legend, table legend, main text, or Methods section).

n/a	Confirmed	
☐	☒	The exact sample size (*n*) for each experimental group/condition, given as a discrete number and unit of measurement
☐	☒	An indication of whether measurements were taken from distinct samples or whether the same sample was measured repeatedly
☐	☒	The statistical test(s) used AND whether they are one- or two-sided<br>*Only common tests should be described solely by name; describe more complex techniques in the Methods section.*
☒	☐	A description of all covariates tested
☐	☒	A description of any assumptions or corrections, such as tests of normality and adjustment for multiple comparisons
☐	☒	A full description of the statistics including central tendency (e.g. means) or other basic estimates (e.g. regression coefficient) AND variation (e.g. standard deviation) or associated estimates of uncertainty (e.g. confidence intervals)
☒	☐	For null hypothesis testing, the test statistic (e.g. *F*, *t*, *r*) with confidence intervals, effect sizes, degrees of freedom and *P* value noted<br>*Give P values as exact values whenever suitable.*
☒	☐	For Bayesian analysis, information on the choice of priors and Markov chain Monte Carlo settings
☒	☐	For hierarchical and complex designs, identification of the appropriate level for tests and full reporting of outcomes
☒	☐	Estimates of effect sizes (e.g. Cohen's *d*, Pearson's *r*), indicating how they were calculated
☐	☒	Clearly defined error bars<br>*State explicitly what error bars represent (e.g. SD, SE, CI)*

Our web collection on statistics for biologists may be useful.

Software and code

Policy information about availability of computer code

Data collection	NA
Data analysis	NA

For manuscripts utilizing custom algorithms or software that are central to the research but not yet described in published literature, software must be made available to editors/reviewers upon request. We strongly encourage code deposition in a community repository (e.g. GitHub). See the Nature Research guidelines for submitting code & software for further information.

Data

Policy information about availability of data

All manuscripts must include a data availability statement. This statement should provide the following information, where applicable:

- Accession codes, unique identifiers, or web links for publicly available datasets
- A list of figures that have associated raw data
- A description of any restrictions on data availability

RNA-seq data from this study is deposited in GEO with Accession code GSE122624. Other data that support the findings of this study are available from the corresponding authors upon reasonable request.

Field-specific reporting

Please select the best fit for your research. If you are not sure, read the appropriate sections before making your selection.

☒ Life sciences ☐ Behavioural & social sciences ☐ Ecological, evolutionary & environmental sciences

For a reference copy of the document with all sections, see nature.com/authors/policies/ReportingSummary-flat.pdf

Life sciences study design

All studies must disclose on these points even when the disclosure is negative.

Sample size	To calculate sample sizes we performed a G-Power analysis on data gathered with blood samples to determine necessary sample size. We included parameters as means and calculate effect size with two-tailed tests.
Data exclusions	No data were excluded.
Replication	All phenotypical data are from biologically different samples (individual patients and healthy controls) as indicated in the manuscript and functional experiments were repeated at least 3 times as indicated in the text.
Randomization	Patients showing active arthritis symptoms were diagnosed by staff rheumatologists following the EULAR/ American College of Rheumatology (ACR) 2010 or ASessment in Ankylosing Spondylitis (ASAS) 2010 criteria upon clinical investigation in the hospital. Healthy control subjects were age- and gender-matched volunteers. Synovial fluid were obtained from patients with an active knee synovitis and an indication for aspiration. All biological samples (blood and synovial fluid) were freshly processed to avoid freeze-thaw effects. Samples were randomly processed.
Blinding	A definite diagnosis was not known at the moment of patient sample processing and was allocated afterwards.

Reporting for specific materials, systems and methods

Materials & experimental systems

n/a	Involved in the study	
☒	☐	Unique biological materials
☐	☒	Antibodies
☒	☐	Eukaryotic cell lines
☒	☐	Palaeontology
☒	☐	Animals and other organisms
☐	☒	Human research participants

Methods

n/a	Involved in the study	
☒	☐	ChIP-seq
☐	☒	Flow cytometry
☒	☐	MRI-based neuroimaging

Antibodies

Antibodies used	All antibody information is shown in the Methods section and Supplementary Table 2.
Validation	Validation was done for all flow cytometry antibodies on both cells and on beads in prior exploratory experiments.

Human research participants

Policy information about studies involving human research participants

Population characteristics	This information is included in Supplementary Table 1.
Recruitment	Patients were recruited at the Rheumatology department of the indicated hospitals.

Flow Cytometry

Plots

Confirm that:

☒ The axis labels state the marker and fluorochrome used (e.g. CD4-FITC).

☒ The axis scales are clearly visible. Include numbers along axes only for bottom left plot of group (a 'group' is an analysis of identical markers).

☒ All plots are contour plots with outliers or pseudocolor plots.

☒ A numerical value for number of cells or percentage (with statistics) is provided.

Methodology

Sample preparation	Human samples were collected and freshly processed for single cell analyses by flow cytometry.
Instrument	BD FACSCanto II and BD FACSAria III
Software	Flowjo 9.8 and 10
Cell population abundance	A minimum of 500 iNKT and 3000 γδ T cells were acquired on a FACS Canto II or Aria III machine (BD) to guarantee accurate calculations of the expression of differentiation markers/transcription factors by these cells.
Gating strategy	Proper gating was based on a Fluorescence minus one controls (FMO) and stainings were performed according to our standardized flow core guidelines (VIB), using dead cell and doublet cell exclusions (as indicated in the text). A detailed gating strategy for relevant subsets is shown in supplementary Figure 1A.

☒ Tick this box to confirm that a figure exemplifying the gating strategy is provided in the Supplementary Information.
